# Regulatory T-Cells and Associated Pathways in Metastatic Renal Cell Carcinoma (mRCC) Patients Undergoing DC-Vaccination and Cytokine-Therapy

**DOI:** 10.1371/journal.pone.0046600

**Published:** 2012-10-31

**Authors:** Adrian Schwarzer, Benita Wolf, Jan L. Fisher, Thomas Schwaab, Sven Olek, Udo Baron, Craig R. Tomlinson, John D. Seigne, Nancy A. Crosby, Jiang Gui, Thomas H. Hampton, Camilo E. Fadul, John A. Heaney, Marc S. Ernstoff

**Affiliations:** 1 Medical Oncology Immunotherapy Group, The Geisel School of Medicine at Dartmouth, Hanover, New Hampshire, United States of America; 2 Section of Hematology/Oncology, The Geisel School of Medicine at Dartmouth, Hanover, New Hampshire, United States of America; 3 Epiontis GmbH, Berlin, Germany; 4 Department of Pharmacology and Toxicology, The Geisel School of Medicine at Dartmouth, Hanover, New Hampshire, United States of America; 5 Section of Urology, Department of Surgery, The Geisel School of Medicine at Dartmouth, Hanover, New Hampshire, United States of America; 6 Section of Biostatistics and Epidemiology, Department of Family and Community Medicine, The Geisel School of Medicine at Dartmouth, Hanover, New Hampshire, United States of America; 7 Immunotherapy Program, The Geisel School of Medicine at Dartmouth, Hanover, New Hampshire, United States of America; 8 Department of Neurology, The Geisel School of Medicine at Dartmouth, Hanover, New Hampshire, United States of America; National Institute of Infectious Diseases, Japan

## Abstract

**Purpose:**

To evaluate CD4^+^CD25^+^FOXP3^+^ T regulatory cells (T_REG_) and associated immune-regulatory pathways in peripheral blood lymphocytes (PBL) of metastatic renal cell carcinoma (mRCC) patients and healthy volunteers. We subsequently investigated the effects of immunotherapy on circulating T_REG_ combining an extensive phenotype examination, DNA methylation analysis and global transcriptome analysis.

**Design:**

Eighteen patients with mRCC and twelve volunteers (controls) were available for analysis. T_REG_ phenotype was examined using flow cytometry (FCM). T_REG_ were also quantified by analyzing the epigenetic status of the FOXP3 locus using methylation specific PCR. As a third approach, RNA of the PBL was hybridized to Affymetrix GeneChip Human Gene 1.0 ST Arrays and the gene signatures were explored using pathway analysis.

**Results:**

We observed higher numbers of T_REG_ in pre-treatment PBL of mRCC patients compared to controls. A significant increase in T_REG_ was detected in all mRCC patients after the two cycles of immunotherapy. The expansion of T_REG_ was significantly higher in non-responders than in responding patients. Methylation specific PCR confirmed the FCM data and circumvented the variability and subjectivity of the FCM method. Gene Set Enrichment Analysis (GSEA) of the microarray data showed significant enrichment of FOXP3 target genes, CTLA-4 and TGF-ß associated pathways in the patient cohort.

**Conclusion:**

Immune monitoring of the peripheral blood and tumor tissue is important for a wide range of diseases and treatment strategies. Adoption of methodology for quantifying T_REG_ with the least variability and subjectivity will enhance the ability to compare and interpret findings across studies.

## Introduction

Although therapies with multi-targeted receptor tyrosine kinase or mTOR inhibitors or agents which block VEGF have made significant inroads in treatment of patients with mRCC, IL-2 therapy remains the only treatment that results in unmaintained sustained complete remissions, albeit in a small percentage of patients [1], [2], [3], [4]. It is therefore important to identify biomarkers which would allow assessment of the probability for patients to benefit from IL-2 therapy. Increasing evidence suggests that immune regulatory pathways, especially regulatory T-cells are the key in limiting the benefits from IL-2 based immunotherapy [5], [6], [7], [8].

We previously reported a study of 18 patients with mRCC who received intranodal vaccination with DC_vacc_ in combination with intravenous high-dose IL-2 and subcutaneous IFN-α2a [9]. With this regimen we observed a surprisingly high objective response rate of 44% (3 complete responses, 5 partial responses, median time to progression of 8 months). In this study we seek to better define the circulating T_REG_ population and associated pathways in these mRCC patients using FCM, methylation specific PCR and whole genome transcriptome analysis.

Naturally occurring CD4^+^CD25^+^ FOXP3^+^ regulatory T-cells (nT_REG_) are a subpopulation of CD4 T-cells capable of suppressing the activation and expansion of T-effector cells, thereby inhibiting the onset of autoimmunity [10]. T_REG_ are characterized by constitutive expression of the IL-2R α-chain (CD25), GITR, CTLA-4, IL-10 and TGF-ß [11], [12]. FOXP3, a member of the forkhead-family of transcription factors is the master regulator of T_REG_ development and function [13], [14], [15]. Loss of FoxP3 leads to functionally deficient T_REG_ and causes fatal autoimmunity [16]. Tumors often induce the expansion of T_REG_ cells and recruit them to the tumor site via soluble factors such as IL10, TGF-ß and VEGF [17]. Hence, cancer patients have significantly more T_REG_ in their blood than healthy humans and show infiltration of the tumor with T_REG_ [18], [19], [20]. IL-2 was initially described as T-cell growth factor and as a consequence used in immunotherapy of RCC and melanoma. However, it has recently been shown that IL-2 therapy substantially expands the number of T_REG_ in cancer patients [18], [21], [22]. Some studies, including ours, suggest that non-responding patients show a higher expansion of T_REG_ following IL-2 based immunotherapy [23]. IL-2 signalling induces the expression of FOXP3 in CD4^+^CD25^+^ cells through binding of phosphorylated STAT5 to the FOXP3 proximal promoter and intron enhancers [22]. Thus, IL-2 is a crucial factor for the development and maintenance of T_REG_ in the periphery [24]. How the tolerance inducing capacity of IL-2 can be reconciled with the anti-tumor effects in 15–25% of patients with mRCC and melanoma remains elusive.

Recently published data shows that FOXP3 and CD25 are not T_REG_-only specific markers. Although new markers are continuing to be found and better gating strategies are proposed (e.g. CD127^low/−^) [25], a clear and concise definition of a FCM-staining panel defining “true” human T_REG_ is still elusive. In addition, FOXP3 is transiently upregulated in human naive T-cells after stimulation [26]. Therefore, it is questionable whether it is possible to distinguish between true T_REG_ and recently stimulated T-cells solely by combined staining for CD25 and FOXP3. Nevertheless, the mainstay for quantification of T_REG_ in the majority of clinical studies has been the enumeration of FOXP3^+^ T-cells with or without inclusion of CD25 via FCM [5], [18]. Due to the lack of a consensus staining panel for T_REG_, published studies have used different markers and gating strategies for the quantification of T_REG_, making comparisons between the studies challenging.

Analyzing the epigenetic status of the FOXP3 locus using methylation specific PCR might be a significant step towards improved quantification of T_REG_. Within the FOXP3 locus exist at least three highly conserved CpG motifs which control FOXP3 expression and are subject to epigenetic modification [27]. One of them, the TSDR (T_REG_ cell specific demethylation region) shows complete and very specific demethylation in T_REG_ [28], [29]. Neither *in vitro* (stimulation with TGFß) generated induced T_REG_ (iT_REG_) expressing FOXP3 nor any other immune cells have a complete demethylated TSDR [29]. This is consistent with data showing that *in vitro* generated T_REG_ display an unstable FOXP3 expression and suppressive potential [28], [30]. In contrast, T_REG_ which are induced *in vivo* by delivery of antigen under tolerogenic conditions show stable long term FOXP3 expression and suppressive potential along with complete demethylation of the TSDR [30]. In summary, the methylation status of the TSDR controls the stability and longevity of FOXP3 expression and is responsible for imprinting of a long lasting suppressive T_REG_-phenotype [27]. It is thus possible to quantify the amount of T_REG_ in a mixed population of cells by determining copy number of demethylated and methylated TSDR by quantitative PCR after bisulfite treatment of genomic DNA. One aim of this study is to compare how well the FCM determined changes in CD4^+^CD25^+^FOXP3^+^ T-cells translate into changes of T-cells which have a long lasting T_REG_ suppressive phenotype due to epigenetic modification of the FOXP3 locus.

With Microarray technology becoming more and more widespread, whole genome transcriptome profiling of PBL and peripheral blood mononuclear cells have been reported to be a potential biomarker surrogate for several medical conditions, including mRCC [31], [32]. In this study we also examined how changes in immune cell subsets translate into changes seen in the PBL gene expression profile and to explore if this approach could be useful for monitoring immune-regulatory pathways during immunotherapy. RNA from patient PBL samples before (n = 17) and after therapy (n = 13) as well as from PBL of controls (n = 9) was hybridized to Affymetrix GeneChip Human Gene 1.0 ST Arrays. The analysis revealed significant enrichment of gene sets and pathways associated with inflammation and counter-regulation in the peripheral blood of mRCC patients compared to healthy controls.

## Materials and Methods

### Ethics Statement

Protocol D0238 (Phase II Clinical Trial with IL-2, IFN-α2a and autologous dendritic cell (DC) tumor vaccination) and Leukapheresis Protocol D9726 were approved by the Dartmouth College Committee for the Protection of Human Subjects (CPHS).

### Patients & Treatment Protocol

As previously reported [23], eligible patients with metastatic RCC were treated on a phase II protocol consisting of IL-2 (Chiron, Inc. CA) administered by continuous infusion at a dose of 18×10^6^ IU/M^2^ for 120 hours. IFN-α 2a (6 MIU, Hofman La Roche, Nutley NJ) was given subcutaneously every other day for 3 doses with the start of each of 5 cycles. DC vaccine (1×10^7^ autologous tumor lysate loaded DCs in 1 ml Lactated Ringer's Solution) was given intra-nodally under ultrasound guidance on the day prior to starting a cycle of IL-2/IFN-α 2a.

### Peripheral blood lymphocyte (PBL) isolation

PBLs were isolated from 18 patients with mRCC and 12 healthy donors (HD). Pre-treatment PBLs from mRCC patients were isolated 9 days before administration of the first treatment. The second isolation (post-treatment) took place 14 days after completion of the 2 induction cycles (33 days from start of therapy). We obtained 12 older healthy donors (mean age 48 years) who signed IRB-approved consent and underwent leukapheresis. Isolation of PBLs was achieved by fractionation of pheresis product on an ELUTRA® Cell Separation System (Gambro BCT, Lakewood, CA). Elutriated PBLs were washed and cryopreserved in 90% autologous serum and 10% DMSO until use.

### Microarrays and RT-PCR

Total RNA was isolated from PBLs using RNAeasy kit (Qiagen, Valencia, CA) according to the manufacturer's instructions. Biotin-labeled cDNA generated from 5.5 µg of total RNA from PBLs of 17 patients pre-treatment, 13 patients post- treatment, and 9 healthy donors was hybridized to the GeneChip® Human Gene 1.0 ST Arrays. Arrays were scanned on an Affymetrix GeneChip Scanner 3000. Microarrays were analyzed using R and Bioconductor [33]. Quality control was performed using ArrayQualityMetrics [34], and arrays were preprocessed with RMA [35]. Differential expression was calculated using LIMMA package with Benjamini & Hochberg multiple testing adjustment. The GSEA algorithm and software has been described elsewhere [36]. For hierarchical clustering and PCA, probesets with an interquartile range >1.8 were selected (n = 1746). Microarray analysis and description was carried out according to Minimum Information About a Microarray Experiment (MIAME) guidelines. The dataset has been deposited in NCBI's Gene Expression Omnibus (http://www.ncbi.nlm.nih.gov/geo/query/acc.cgi?acc=GSE34465) and is accessible through GEO Series accession number GSE34465.

### FCM Staining

Fluorochrome conjugated anti-human antibodies were purchased from the indicated suppliers. CD3, CD4, CD8, CD45RA, CD25 from Beckman Coulter (Fullerton, CA): GITR and, CTLA-4 from BD Pharmingen (San Jose, CA). Intra-nuclear FOXP3-Staining was carried out with the Biolegend FOXP3-Kit (San Diego, CA) according to manufacturer's instructions. All samples were acquired on a FACS Canto (Becton Dickinson, San Jose, CA) and analyzed with FlowJo software (Tree Star Inc. Ashland, OR). To determine cells positive for respective markers, we set the gates at the <1% level of the respective isotype controls with appropriate FMO (fluorescence minus one) staining combinations.

### Definition of FCM gates

Our FCM data is from multi-parameter staining of PBL samples with a combination of CD3, CD4, CD25 and intra-nuclear FOXP3. For comparisons to the PCR data (see below) we define T_REG_ based on two gating strategies relevant to the published T_REG_ data from human immunotherapy trials. The gating strategies employed for the T_REG_ are shown in Supplementary Fig S5. Lymphocytes are pregated on CD3^+^CD4^+^ T-helper cells. The FOXP3^+^ events within the CD3^+^CD4^+^ population are defined as single positive regulatory T-cells and subsequently referred to as **SP-T_REG_** (**SP-T_REG_ = CD3^+^CD4^+^FOXP3^+^**). The CD25^+^FOXP3^+^ events within the T–helper cell population are defined as double positive regulatory T-cells and will be called **DP-T_REG_ (DP-T_REG_ = CD3^+^CD4^+^CD25^+^FOXP3^+^)**. The FCM determined amount of T_REG_ is presented as a percentage with a numerator of SP- or DP-T_REG_, and a denominator of the total lymphocytes (PBL) or the CD3^+^ T-cell population. These proportions are compared to the %TSDR of total lymphocytes or %TSDR of CD3-T-cells as determined by methylation specific PCR. The absolute number of Tregs were quantified by a complete blood count performed the day of the elutriation of the PBL. Absolute Treg numbers were calculated by multiplying the proportion of DP-T_REG_/PBL as quantified by FCM with the absolute lymphocyte count/ul from the blood count. For analysis of the surface markers GITR, CTLA-4, CCR7 and CD45 isoforms cells were pregated on lymphocytes and then 1–2% of these cells which were CD4^+^ and had the highest expression of CD25 were selected for analysis. This gating strategy resulted in a subset of cells that was nearly 100% FOXP3^+^ T-cells (Supplementary Fig S1A). This population will be subsequently referred to as **CD4^+^CD25^high^ T-cells**. The gating strategies employed are shown in Supplementary Fig S6.

### Methylation specific Real Time-PCR for FOXP3 and CD3

Bisulfite-conversion was performed applying the EpiTect Bisulfite Kit (Qiagen) using 1–2 ug of genomic DNA and following the suppliers' recommendations. Quantification of Treg and overall T-cells by means of epigenetic qPCR analysis was carried out as described previously [37], [38].

### T_REG_ functional assay

The suppression assay was carried out as previously described [39]. Briefly, the CD4CD25^high^ fraction and CD4CD25^−/low^ responder T-cells were isolated from PBL using the T_REG_ Isolation Kit from Miltenyi Biotec (Auburn, CA). CD4+ T cells were negatively isolated. CD4^+^CD25^high^ T-cells were isolated from the CD4+ cells by direct labelling with anti-CD25 microbeads (Miltenyi Biotec) followed by separation into CD25^high^ T_REG_ (>95% purity) and a CD25^−/low^ fraction (responder cells). T Cell Activation/Expansion Beads (Miltenyi Biotec) were prepared according to manufacturer's instructions. 2.5×10^4^ CD4+ CD25^−/low^ responder T cells were combined with varying numbers of CD4^+^CD25^high^ T_REG_ cells and stimulated with 50,000 T Cell Activation/Expansion Beads per well then cultured in triplicate for 5 days at 37°. On day 5, cultures were pulsed with [^3^H] Thymidine (Perkin-Elmer, Boston, MA) for the last 16 to 18 hours of culture, harvested, and incorporated radioactivity measured.

% Suppression was calculated as: 
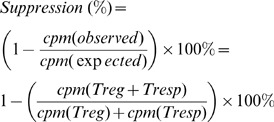



### Statistical Analysis

Statistical analysis and visualization was done using R and SigmaStat Software. Data are expressed as mean and standard deviation (SD) for absolute numbers and percentage and depicted as scatter plots with the arithmetic mean indicated as a line, or as standard Box and Whiskers Plots. These plots indicate the 25^th^ and 75^th^ percentile (bottom and top box edges), median value (line in box) and the low and high values (error bars). Statistical analysis was performed by testing for normality and equal variance and using Student's *t* test to assess differences between the different study groups. Where data did not have equal variance, t-test with Welshs correction was used. *P≤0*.05 was considered significant.

## Results

### Clinical results

The clinical results of the study have been previously published [23]. Briefly, eighteen patients with advanced metastatic mRCC (13 males, 5 females) were enrolled in the study. All patients received up to 5 cycles intranodal vaccination with autologous tumor lysate pulsed dendritic cells combined with high dose IL-2 and IFN-alpha. The patient characteristics and clinical outcomes are summarized in Supplementary table 1. Overall objective clinical response rate was 44% with three long lasting complete responses.

### FCM analysis of healthy donor and pre and post-treatment patient T_REG_ populations

**Figure 1:**
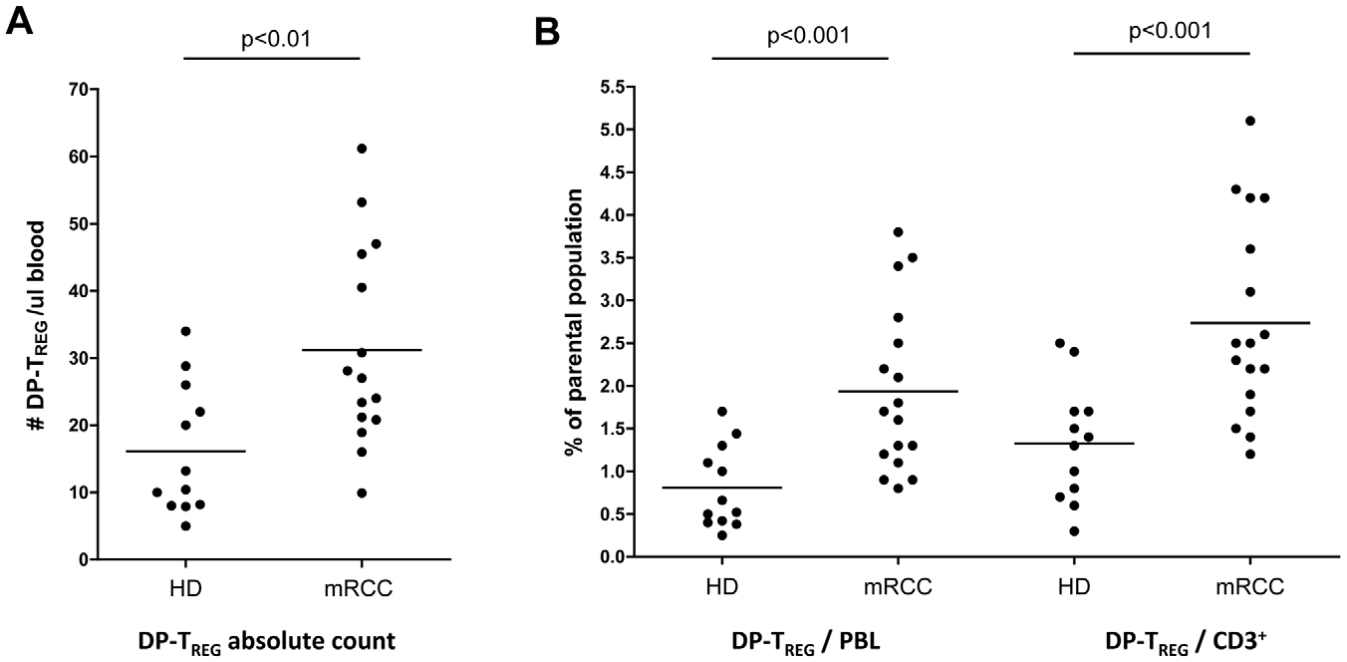
nT_REGs_ are increased in the peripheral blood of RCC patients. (A) Absolute numbers (HD = 12, mRCC = 15, for patients #3, #6 and #18 a complete blood count from the day of the elutriation was not available) and (B) frequencies of T_REG_ in the circulation of HD (n = 12) and patients with mRCC (n = 17, all but patient #15).

Flow cytometry analysis confirmed that patients with mRCC had significantly higher absolute number of DP-T_REG_ **(DP-T_REG_ = CD3^+^CD4^+^CD25^+^FOXP3^+^)** in their peripheral blood than the 12 healthy donors that were available for the analysis (**HD 16**±10 cells/µl vs **mRCC 31**±15 cells/µl; p<0.01. Fig. 1A). The proportion of DP-T_REG_ in both the total PBL and in the T-cell compartment was significantly elevated in mRCC-patients *(%DP T*_REG_ *of PBL*: **HD 0.81**±0.48% vs **mRCC 1.94**±0.96% p<0.001, and %*DP T*_REG_ *of CD3+*: **HD 1.30**±0.69% vs **mRCC 2.74**±1.16% p<0.001 Fig. 1B), corroborating a disease effect on this cell population.

**Figure 2:**
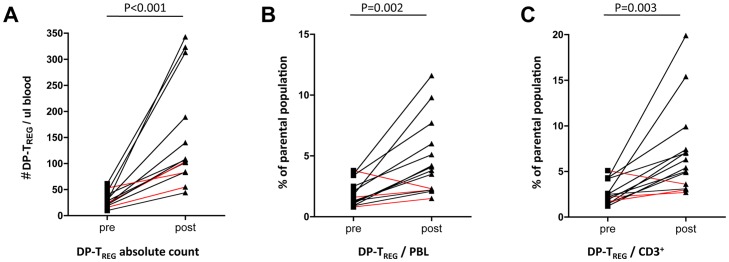
Frequencies and absolute numbers of T_REG_ in the circulation of patients before therapy (Pre) and 14 days after the completion of the two induction cycles (Post) (n = 14, all but patient #3,6,15,18). Pre and post of each individual patient are connected by a line. Red lines indicate complete responders. (A) absolute T_REG_ numbers per ul blood, (B) proportion of DP-T_REG_ within the PBL and (C) within the CD3^+^ compartment.

In order to determine a treatment effect in our patient population, we compared levels of DP-T_REG_ before therapy (Pre) and 14 days after completion of the two induction cycles (Post). We observed a significant increase of T_REG_ absolute numbers in the blood post treatment (**Pre 30**±15 cells/µl vs **Post 150**±102 cells/µl; p<0.001, Fig. 2A). The frequency of T_REG_ cells within the total lymphocyte and within the CD3^+^ T-cell compartment increased significantly, showing that the population of DP-T_REG_ was expanded relative to other immune (effector) cells: (*%DP-T_REG_ of PBL:* **Pre 1.9**±1.0 vs **Post 4.7**±3.1 p = 0.002, Fig. 2B and *%DP-T_REG_ of CD3^+^:* **Pre 2.7**±1.2 vs **Post 7.2**±5.0 p = 0.003; Fig. 2C). Thus, the frequencies of circulating DP-T_REG_ had on average almost tripled after two cycles of IL-2 based immune therapy.

**Figure 3:**
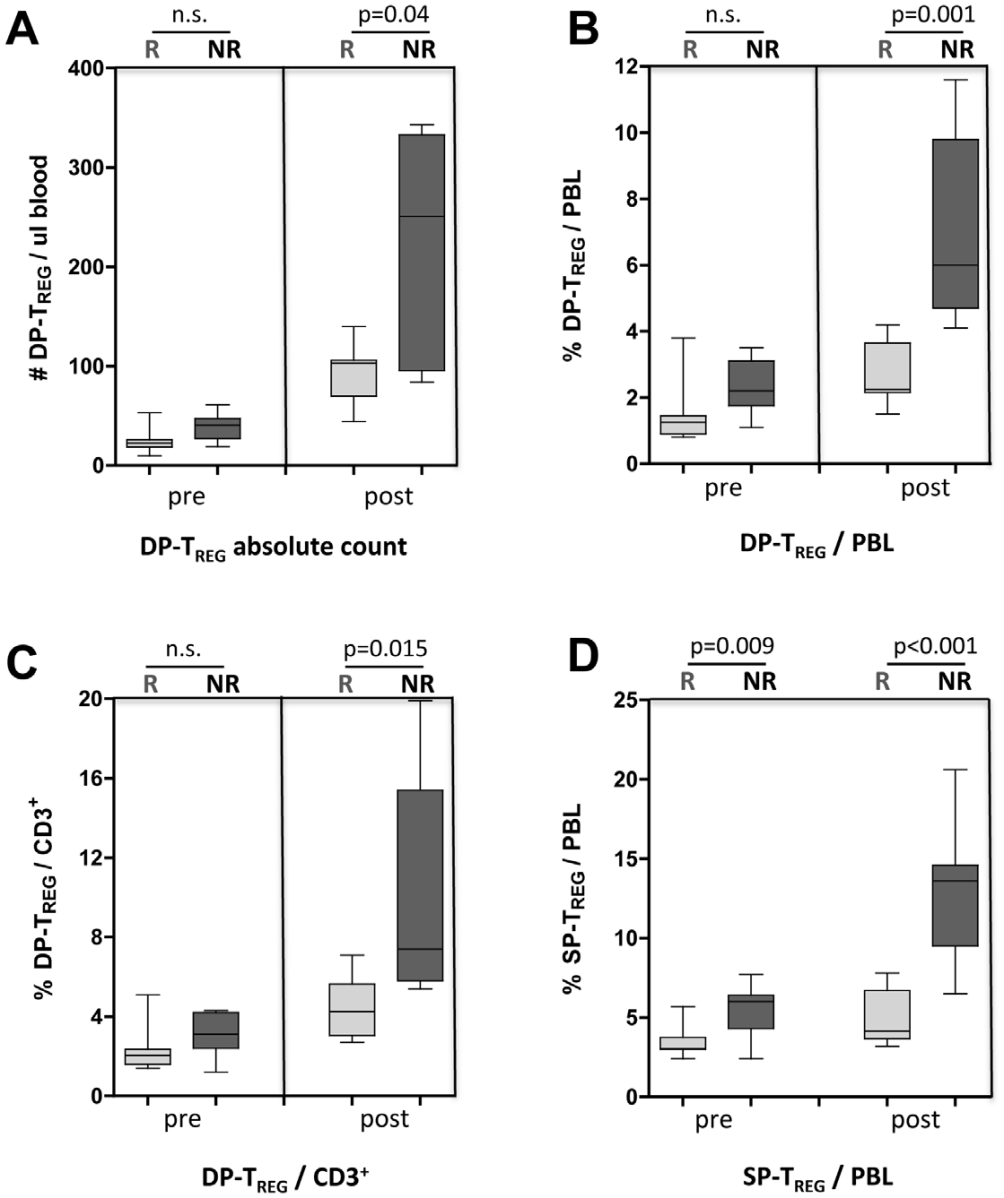
Box and Whiskers Plots of the absolute T_REG_ number for responders and non-responders pre and post therapy (A). Comparison of proportions of DP-T_REG_ of PBL and CD3^+^ in the circulation of responders and nonresponders Pre and Post-treatment, respectively (B) & (C). Proportion of SP-T_REG_/PBL for R and NR pre and post therapy (D). Included patients: R PRE and POST: #2,4,7–9,12,16,17; NR PRE:#1,3,5,6,10,11,13,14,18; NRPOST: #1,5,10,11,13–15.

Patients were then divided into responders (complete: CR = 3 and partial response: PR = 5) and non-responders (stable disease: SD = 7 and progressive disease: PD = 3) based on National Cancer Institute's Response Evaluation Criteria in Solid Tumors. Pre-treatment, no statistically significant differences could be found in the numbers and proportions of DP-T_REG_ in these two groups. However, absolute numbers and frequencies of DP-T_REG_ post treatment were significantly higher in non-responders (NR) than in responding patients (R) (*absolute numbers*: **NR 227±**115/µl vs **R 93±**31/µl p = 0.04, Fig. 3A; *%DP-T_REG_ of PBL:* **NR 7.0±**2.8 **vs R 2.7±**1.0 p = 0.001, Fig. 3B and *%DP-T_REG_ of CD3:* **NR 10.1±**5.5 vs **R 4.4±**1.6 p = 0.015, Fig. 3C). Strikingly, patients achieving complete and durable remissions showed the least expansion, or even a reduction of the proportion of DP-T_REG_ (marked in red in Fig. 2A–C). Analysis of SP-T_REG_ (**SP-T_REG_ = CD3^+^CD4^+^FOXP3^+^**) populations in responders and non-responders revealed differences for both baseline and post therapy comparisons. Responders had a significantly lower proportion of SP-T_REG_ for both timepoints (*%SP-T_REG_ of PBL:* **Pre: NR 5.4±**1.6 vs **R 3.5±**1.0 p = 0.009 and **Post: NR 13.0±**4.4 vs **R 5.0±**1.8 p<0.001, Fig. 3D). Lowering the gating cutoff for CD25 led to results approaching those of the SP-gating strategy for the pre treatment comparison between responders and non-responders (data not shown). This illustrates that setting the gates on continuous markers such as CD25 can be difficult even with proper isotype controls (Supplementary Fig. 1A) and may lead to different conclusions based on the same dataset.

We further characterized the T_REG_ cell population in the patients by evaluating the expression of CTLA-4, GITR, CCR7 and CD45RA within the CD4^+^CD25^high^ compartment (Supplementary Fig S2A). CTLA-4 could be found consistently on the surface of about 80% of the CD4CD25^high^ population of healthy donors as well as in patients before and after therapy. GITR, however, was only detected on a minor fraction of CD4CD25^high^ cells. Most CD4CD25^high^ were found to belong to the central memory subtype in both patients and healthy donors (*%CCR7^+^ CD45RA^−^:* **Pt 63.8**±16.4 vs **HD 56.6**±33.4; ns; Supplementary Fig S2B). About 10% were naive T_REG_ (*%CCR7^+^ CD45RA^+^:* **Pt 9.8**±6.8 vs **HD 7.2**±5.6; ns). In summary, no significant differences in the surface phenotype of CD4^+^CD25^high^ T-cells in untreated mRCC patients and healthy donors could be detected using these markers.

In further analysis, the expression of CTLA-4 and GITR did not significantly differ between pre and post-therapy T_REG_. However, within the CD4^+^CD25^high^ compartment we detected a significant treatment related shift towards naive (CD45RA^+^ CCR7^+^) T-cells (**Pre 6.3±**5.7% vs **Post 24.8±**11.37% p<0.001; Supplementary Fig S2B) at the cost of the central memory (CD45RA^−^CCR7^+^) CD4^+^CD25^high^ T-cells. In analysis of response related differences, the expression of CTLA-4 and GITR and the distribution between memory and naive CD4^+^CD25^high^ T-cells did not differ between CD4^+^CD25^high^ T-cells from these two groups (data not shown), suggesting that the difference between responders and non-responders are different frequencies of T_REG_, not different phenotypes of the regulatory cell population.

### Functional suppressive ability of patient T_REG_ cells

The suppressive ability of enriched CD4^+^CD25^high^ T-cells was analyzed for six patients pre and post-treatment cells, as well as for 4 healthy donor controls. T_REG_ -Suppression assays (Supplementary Fig S3) revealed that the CD4^+^CD25^high^ T-cells from mRCC patients were functional and efficiently inhibited proliferation of CD4^+^CD25^−^ responder cells with no significant difference from the CD4^+^CD25^high^ T-cells of healthy donors.

### Relationship of methylation specific PCR results and FCM data

In the PCR method T_REG_ were quantified by determining the proportion of demethylated TSDR alleles compared to methylated TSDR alleles in the patient PBL samples. Genomic DNA was treated with bisulfite and the differently methylated TSDR were amplified with methylation specific primers in a quantitative PCR [38]. Twelve patient samples (6 responders, 6 non-responders) were available for pre and post-treatment comparison. For determination of the frequency of CD3^+^ T- cells within the sample a similar methylation specific PCR was performed interrogating the methylation state of the CD3 locus. Results of the PCR analysis are reported as % dTSDR/PBL and % dTSDR/CD3^+^ (d = demethylated) reflecting the proportion of T_REG_ of all cells in the elutriated sample (lymphocytes) and the proportion of T_REG_ within the CD3^+^ T-cell compartment, respectively. The latter was obtained by normalizing the number of demethylated TSDR alleles to the number of demethylated CD3 alleles.

**Figure 4:**
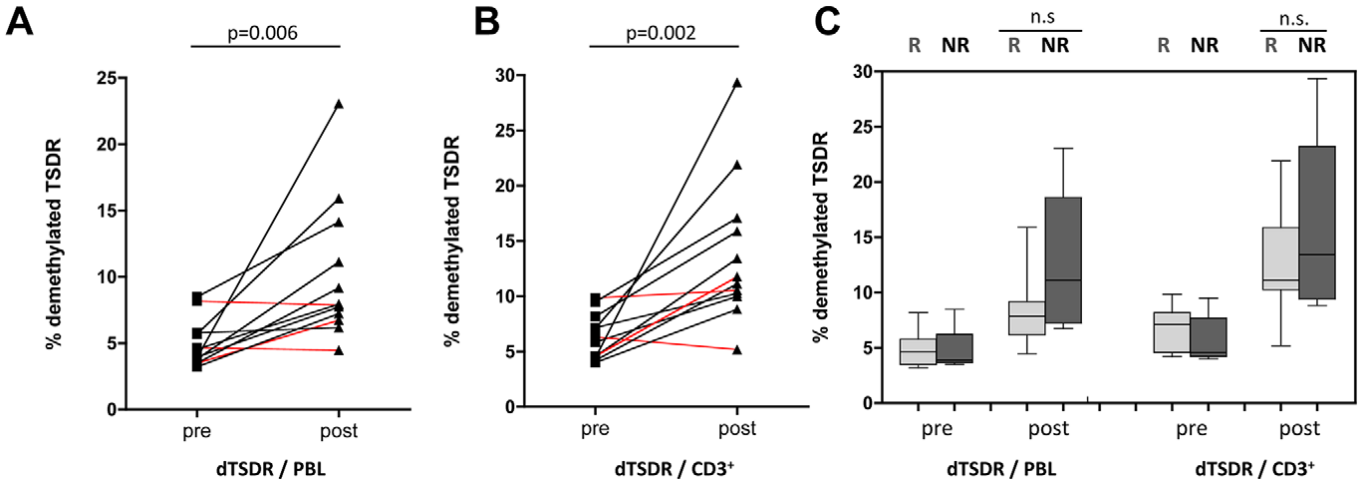
Methylation specific PCR for 12 patients with pre and post samples available (#1, 2,4,5,7–10,13,14,16,17). (A) Treatment related results of (% dTSDR/PBL and (B) %dTSDR/CD3^+^: % demethylated alleles of T_REG_ cell specific demethylation region within the lymphocyte and T cell population respectively. Red lines indicate complete responders (C) Response related results of methylation specific PCR for pre and post-therapy T_REG_ (R = 6, NR = 6).

The PCR results corroborated the FCM findings for a treatment effect: post-treatment samples had an average of more than 2 fold the T_REG_ of the pre-treatment samples for both the lymphocyte and CD3 PCR quantification (*% dTSDR/PBL:* **Pre 4.9**±1.8% vs **Post 10.1**±5.2% p = 0.006, Fig. 4A; *% dTSDR/CD3^+^:* **Pre 6.3**±2% vs **Post 13.8**±6.8% p = 0.002; Fig. 4B). Only two patients (#4 and #8) showed a reduction in the absolute number of methylated TSDR alleles, and strikingly, both were complete responders. The third CR (#17) had the lowest absolute number of dTSDR alleles at baseline and showed a moderate increase after the therapy (Fig. 4A). A fourth patient (#16) who demonstrated stable dTSDR/PBL levels exhibited a near complete response, however the response was short-lived (TTP 7 months).

**Figure 5:**
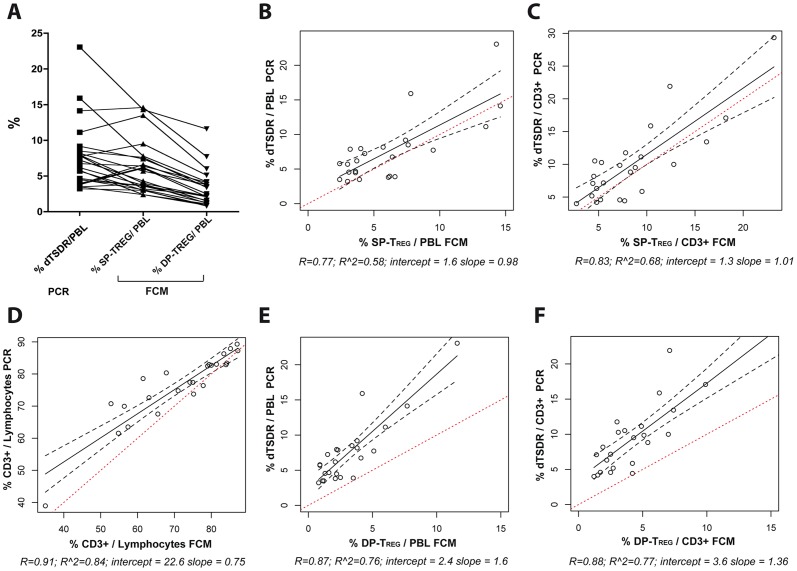
T_REG_ estimates obtained by methylation specific PCR and two FCM gating strategies for 24 samples (12 pre, 12 post). (A) Measurements for the same sample are connected by a line. (B–F) Linear regression between the results of methylation specific PCR for the TSDR locus and defined FCM populations. Solid black: regression line; Black dotted: 95% confidence interval of the regression line; Red dotted: Id-line with a slope of 1.0 and an intercept of 0. In subscript numerical results of the linear model.

Overall, responding patients showed a lower amount of dTSDR in their samples after therapy. However, it failed to reach statistical significance due to limited numbers of patients available for the analysis (*% dTSDR of PBL:* **R Post 8.4**±3.5% vs **NR Post 12.6**±6.5 p = 0.25, Fig. 4C). The PCR based method generally reported a higher proportion of T_REG_ cells than the respective T_REG_ proportions as determined by FCM (Fig. 5A). Particularly FCM gating on DP-T_REG_ cells seemed to underestimate the proportion of regulatory T-cells as quantified by PCR by a factor of more than 2. Gating on the SP-T_REG_ -cells led to numerical results which matched the values obtained by PCR more closely (*% dTSDR* **7.5**±4.5; *SP-T_REG_* **6.1**±3.7; *% DP-T_REG_* **3.2**±2.6; n = 24; Fig. 5A). This suggests that by conservative FCM gating on the CD25^+^FOXP3^+^ DP-T_REG_ population, a significant portion of functionally stable regulatory T-cells may not be taken into account.

Linear regression revealed an overall high degree of correlation between all the FCM gating strategies and the respective PCR results (Fig. 5 B–F). Quantification of CD3^+^ T-cells within the lymphocyte population by methylation specific PCR-analysis of the CD3-locus compared to the proportion of CD3^+^ by FCM achieved a correlation coefficient of 0.91 (Fig. 5D). Linear regression between the PCR results and % DP-T_REG_ resulted in regression lines substantially above the id-line (Fig. 5 E–F), again indicating that FCM in our hands, underestimated the proportion of T_REG_ within a mixed population of cells. Despite the differences in absolute values for the two methods, the FCM results from the DP-T_REG_ gating (Fig. 5 E–F) were better correlated with the PCR results than FCM gating on SP-T_REG_ (Fig. 5 B–C). In summary, gating on the CD4^+^CD25^+^FOXP3^+^ DP-T_REG_ led to quantification of T_REG_ different in absolute value but with a high prediction confidence for the relative proportion of stably suppressive regulatory T-cells as quantified by TSDR-PCR.

### Analysis of enrichment of gene sets associated with immune regulatory pathways

**Figure 6:**
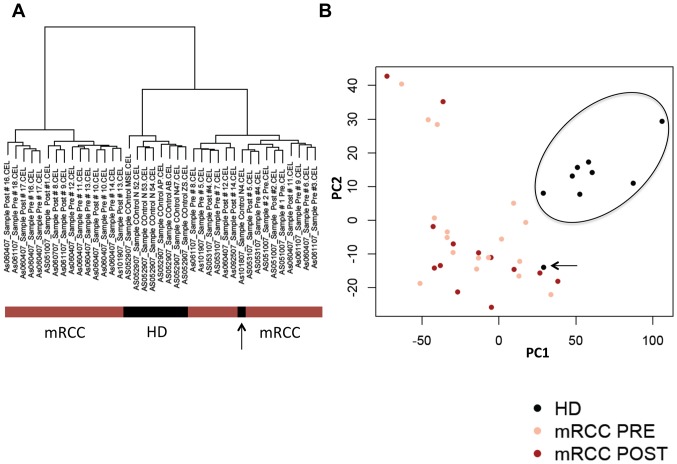
Ward-dendrogramm obtained by unsupervised hierarchical clustering of patients and control (HD) samples using Manhattan distance (A), Principle Components Analysis. Encircled are the healthy controls with one outlier (arrow) (B). Each point represents one microarray sample. The plot was obtained by projecting the samples from the feature space onto the first three principle components, which cover about 50% of the total variance in the data.

RNA from patient samples pre (n = 17; #1–14 and #16–18) and post-therapy (n = 13, #1,#2,#4,#5,#8–14,#16,#17) as well as nine available controls was processed and hybridized to Affymetrix GeneChip Human Gene 1.0 ST Arrays. For unsupervised analysis, 1700 probesets with the largest variance were selected. Hierarchical clustering and principle components analysis (Fig. 6A and B) demonstrated that all HD but one formed a distinct cluster clearly separated from the patient samples. An obvious clustering of the patient samples based on grouping by the treatment related variables Pre, Post or Responder, Non-responder was not observed (Supplementary Fig S4). This result was reproducible when the variance based gene filter (interquartile range, IQR) was set higher to select fewer genes or lower for more genes (data not shown).

**Figure 7:**
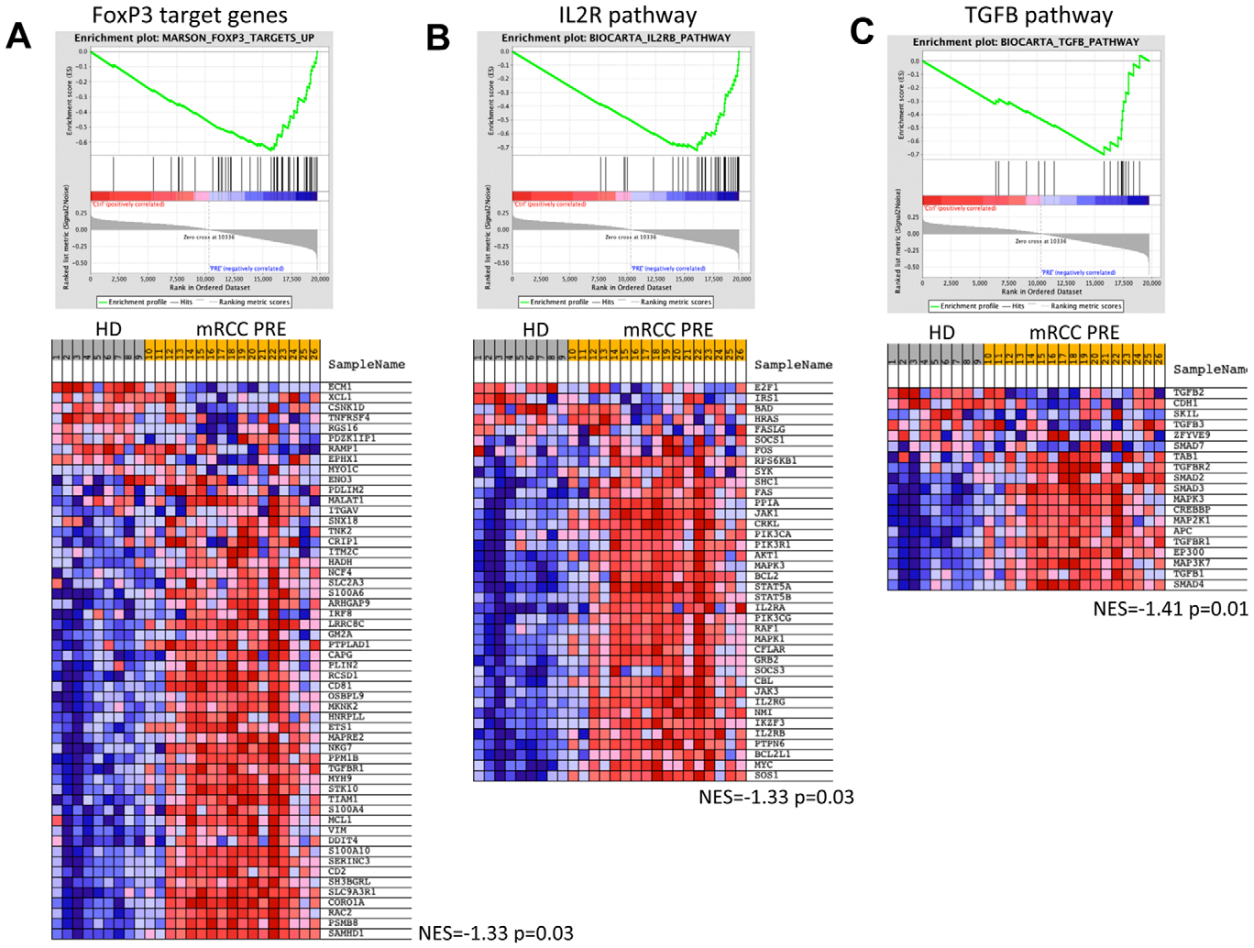
Gene Set Enrichment Analysis for 9 healthy donors (grey) and 17 mRCC samples pre-treatment (yellow). Shown are representative regulatory pathways that were ranked among the top 50 gene sets upregulated in the mRCC samples. (A) Marson FOXP3 target genes (p = 0.03), (B) IL2R pathway (p = 0.03), (C) TGFB pathway (p = 0.01).

We focused our analysis on pathways commonly associated with T_REG_ and immune-regulation, using Gene Set Enrichment Analysis [36]. From more than 3000 curated gene sets stored in the MySig Database (C2.All.V3.0/Broad Institute, MIT) we searched for gene sets that matched one of the following terms: regulatory, FOXP3, CTLA-4, TGF-ß, SMAD, IL2 or T-cell signal transduction. This selection was done to increase test power and reduce irrelevant discoveries by testing thousands of gene sets stored in the database not related to the immune system. A list consisting of 16 gene sets matching these terms (Supplementary table 2) was compiled and used to test for enrichment in HD vs pre-treatment patient phenotypes. Significantly enriched in mRCC patients (Fig. 7, Supplementary Table 3) were the Biocarta TGF-ß pathway (rank 1, p = 0.013, FDR = 0.17), both FOXP3 target gene sets from Marson et al (Ref; rank 2 and 3, p = 0.04, FDR = 0.14 and 0.17) and the Biocarta IL2R pathway (rank 4, p = 0.03, FDR = 0.137). Also enriched were the Biocarta CTLA-4 inhibitory pathway (rank 7, p = 0.04, FDR = 0.133) and TCR pathway (rank 6, p = 0.04, FDR = 0.013). Similar comparisons of pre vs post and responders vs non-responders, applying the selected gene sets, showed no relevant differences related to treatment or response to treatment.

An unsupervised approach testing all available gene sets (n≈3000) found the previously mentioned immune regulatory signatures among the top 50 of all tested gene sets, with TCR and FoxP3 ranking 2 and 6, respectively. Furthermore, gene sets related to mTOR-activation, cell cycling and receptor tyrosine kinase signaling were found to be highly enriched in the mRCC patient samples. Again, no gene sets or individual genes were robustly differentially regulated for Pre vs Post or R vs NR.

## Discussion

In recent years it has become evident that tumors actively evade eradication by the immune system by several mechanisms. T_REG_ are a notable one of these, and are now considered a major obstacle towards successful immunotherapy. Several studies have addressed the role of T_REG_ in the clinical setting of cytokine therapy, particularly in melanoma and mRCC [5], [18], [21], [40]. In this analysis, we determined the impact of combined vaccination and high dose cytokine therapy on number and function of T_REG_ cells *in vivo* employing three different methodological approaches currently available for immune monitoring of clinical trial samples.

Our study confirms previous reports that mRCC patients have higher numbers of circulating T_REG_ than healthy controls. We demonstrated that the expanded T_REG_ cells in mRCC patients are functional and further characterized them as similar to those in healthy individuals with regard to expression of CTLA-4 and GITR, as well as naive and memory phenotype.

DC-vaccination combined with high dose IL-2 and IFNα increased the absolute number and percentage of circulating T_REG_ significantly. In the course of therapy responding patients exhibited a significantly smaller expansion of their T_REG_ cells. The group of patients enrolled in this trial were mainly in the intermediate MSKCC category. This homogeneity of patient stage should limit a possible influence of the overall tumor burden on the outcomes observed. Our results corroborate previous findings of at least three other groups. Jensen et al. looked for FOXP3^+^ cells in mRCC tumor core biopsies in patients undergoing IL-2 therapy [40]. They showed that intra-tumoral FOXP3^+^ regulatory immune cells significantly increase during IL-2-based immunotherapy. Patients with high expansion of FOXP3^+^ cells in biopsy specimens had a significantly worse prognosis than patients with only a moderate rise in the FOXP3^+^ cell numbers. Cesana et al. reported a poor outcome for patients with a very high number of T_REG_ in their blood after IL-2 based immunotherapy as well [18]. Rosenberg et al evaluated the outcome from four clinical trials employing adoptive T-cell transfer combined with various conditioning regimes and found that the levels of endogenous CD4+ FoxP3+ T-cells are inversely correlated with outcome [5].

Some studies have reported that higher numbers of T_REG_ in the blood at the beginning of therapy influenced the outcome [41]. Although we found significantly lower pre-treatment T_REG_ proportions in responding patients based on the SP- T_REG_ FCM gating (Fig. 3D), it was not statistically confirmed based on the DP-T_REG_ FCM gating strategy or the PCR method. Notably, in our study the patient who had the highest pre-treatment T_REG_ proportion of all the patients (#8) exhibited a long lasting complete remission. Strikingly, this was the only patient who had a significant reduction in the proportion of T_REG_ as determined by FCM and PCR upon therapy, highlighting that minimizing the level of T_REG_ expansion under immunotherapy might be more important for objective clinical responses than the pre-treatment T_REG_ levels. Overall, our data support the use of quantifying T_REG_ as a surrogate marker for monitoring immunotherapy in patients, and highlight the prognostic importance of T_REG_ expansion under immunotherapy.

In light of these results, methods that allow reliable and consistent measurement of stable suppressive T_REG_ across studies are essential. The recently developed methylation specific PCR-based method [38] to quantify T_REG_ by determining the amount of demethylated (T_REG_ specific) and methylated (all other immune cells) TSDR sequences in a sample may help achieve this goal. We consistently found a lower proportion of DP-T_REG_ by FCM compared to the TSDR PCR method, which likely resulted from the presence of a significant amount of true T_REG_ in the CD25^low/−^ population which was excluded by conservative CD25+ FCM gating, but detected by the PCR method. Gating only on FOXP3+ T-cells (SP-T_REG_), as for example used by Rosenberg et al [5], results in FCM T_REG_ values that match the results of the PCR better, but the “noise” introduced by inclusion of recently activated FOXP3^+^ T-cells which are not true T_REG_ is reflected in a lower correlation of SP-T_REG_ flow cytometry and PCR results. However, even though Rosenberg et al found, with a far larger number of patients than our study, a clear association between clinical response and T_REG_ levels, they also found that the levels of T_REG_ had a poor prediction confidence for clinical outcome using logistic multivariable regression [5]. The authors concluded that T_REG_ levels are not useful as significant predictor of response to immunotherapy [5]. However, in this study we show that gating on CD4^+^FoxP3^+^ cells has a lower prediction confidence for the true levels of T_REG_ as gating on CD4^+^CD25^+^FoxP3^+^. The noise inherent to the CD4FoxP3 method might have contributed to the lack of predictive power found by Rosenberg et al. It is therefore neccessary to establish a more precise and reproducible measurement of T_REG_ before final conclusions can be drawn whether T_REG_ can serve as a useful biomarker or not.

Most studies to date have only reported FCM results for T_REG_ quantification, which brings into question the contribution of (IL-2) activated T-cells and transiently induced T_REG_ to these results, as some of these cells express CD25 and FOXP3 without actually being stable functional T_REG_ [27]. The methylation specific PCR results from our study show that IL-2 based immunotherapy leads to a substantial expansion of T-cells with a demethylated TSDR, i.e. T_REG_ which have a long lasting suppressive phenotype due to epigenetic modification of the FOXP3 locus. More substantial expansion of T_REG_ post- therapy in non-responding patients than in responding patients observed within the FCM data is supported by the PCR method results, although it does not reach statistical significance. This can be explained by a lower patient sample number available for PCR analysis (12 patients, Fig. 3) compared to FCM (18 patients, Fig. 2). Whether IL-2 therapy expands the peripheral pool of T_REG_ or enhances thymic output, or both, remains to be determined. Results of studies with greater patient numbers will be needed to establish whether the epigenetic method for T_REG_ quantification is superior to predict patient outcome compared to FCM based strategies. In our study, incorporating additional FCM markers of T_REG_ (CTLA4, GITR, etc.) did not allow further discrimination of treatment related or response related differences between patients. Our results support the use of the methylation specific PCR method because it circumvents much of the variability and subjectivity of the FCM method of T_REG_ quantification.

Genomewide transcriptional profiling of cell samples of patients is being adopted as a mainstay in the search for biomarkers predicting outcome or monitoring therapeutic response. We applied microarray analysis to PBL patient samples and healthy controls, and for this study focused on the impact of mRCC and immunotherapy on pathways associated with T_REG_ and immunosuppressive cytokines using GSEA. Analyzing gene sets rather than single (“significant”) genes has been proven to be a valuable tool to compare clinical microarray datasets, due to the high variation inherent in patient derived expression profiles, leading to lack of reproducibility between studies [36]. In GSEA the overall behaviour of groups of biologically related genes is assessed without arbitrary cut offs, which is more sensitive and less error prone than the single-significant gene approach [42].

In our study, expression profiles of mRCC patients were globally different from the healthy donors assigning them to a distinct cluster in PCA and unsupervised hierarchical clustering. This makes it possible to assign a sample to the patient or control cohort based on the gene expression signature. We queried more than 3000 gene sets available from public databases and found gene sets related to cell activation, cell cycling and receptor tyrosine kinase signaling were highly enriched in the mRCC patient samples, suggesting a state of enhanced activation of the immune system. A priori defined gene sets associated with regulatory T-cells comprising the FOXP3, TGF-ß, IL-2 and CTLA-4 pathways were highly upregulated in the patient samples. In contrast to the large difference between healthy donors and mRCC patients, we failed to detect robustly differentially regulated genes or gene sets between Pre and Post therapy nor between responding and non-responding patients using the preselected immune-regulatory gene sets. A possible explanation why the changes in the T_REG_ compartment during therapy and between R and NR did not translate into readily dectectable changes in the expression profiles is that T_REG_ comprise a relatively small subset of immune cells. It is highly likely that treatment related changes in other immune cell subsets e.g. NK cells and T-cells [23], that make up a considerably larger fraction of PBL, mask the changes occuring within the T_REG_ population. Furthermore, the considerable variance in the expression profiles of patients from different genetic background and clinical course as well as limitations in sample size restricted the power to detect possible differences. For a more thorough analysis and discussion of this dataset see Wolf et al. 2012 [43]. Larger studies across different cancer types are needed to clarify whether “cancer type specific” cell expression profiles do exist in PBMC and if they are useful for screening or monitoring (immune) therapy.

In the clinic, cytokine therapy treatment for mRCC patients has been replaced by small molecule inhibitors or antibodies like Sorafenib/Sunitib which offer a higher response rate and less adverse effects. However, these treatments fail to induce long lasting (complete) remissions, which have been observed in a limited number of patients treated with immunotherapy. Combined multi-modality treatment strategies for cancer that incorporate ways to minimize T_REG_ and other mechanisms of suppression, and that elicit tumor specific immunity using vaccines, cytokines or adoptive cellular approaches remain an attractive therapeutic approach. Monitoring treatment related changes in T_REG_ in peripheral blood and tumor tissue will continue to be important for a wide range of diseases and treatment strategies, and “standardization” of T_REG_ quantification would be beneficial.

## Supporting Information

Figure S1 The FCM plot, pre-gated on lymphocytes, shows that within the CD25^high^ population nearly 100% of the cells are also FoxP3^+^(A), Linear regression between CD4+CD25^high^ T-cells and CD4^+^CD25^+^FOXP3^+^ DP-T_REG_ both measured by Flow Cytometry reveals a correlation of R = 0.91 between the two different populations(B).
(TIF)

Figure S2 Expression of surface molecules within the CD4^+^CD25^high^ compartment. (A) Expression of CTLA-4, and GITR did not differ between healthy controls and mRCC patients. (B) Distribution of T-cells belonging to central memory, effector memory or naive phenotype within the CD4^+^CD25^high^ T-Cell compartment in HD and mRCC patients before and after therapy.
(TIF)

Figure S3 T_REG_ suppression assay. CD4^+^CD25^−^ T-cells were mixed with CD4^+^CD25^high^ regulatory T-cells and stimulated with T-cell activation/expansion beads. Proliferation was measured by [^3^H]-Thymidine incorporation. Patients with pre and post samples available for the assay were: #1,2,5,9,13,16.
(TIF)

Figure S4 Principle components analysis of the patients' microarray samples with respect to the variables Non-responder, Responder, Pre and Post
(TIF)

Figure S5 FCM gating strategies for (A) DP- and (B) SP-T_REG_ as described in materials and methods. CD3 plot is pre-gated on lymphocytes by scatter.
(TIF)

Figure S6 FCM gating strategies for CTLA4: isotype control in gray, CTLA4 Ab solid black line(A), CCR7/CD45RA T memory cell gating strategy(B). CD3 plot is pre-gated on lymphocytes by scatter.
(TIF)

Table S1 Clinical data for enrolled patients (n = 18). Abbreviations: M = male, F = female, cc = clear cell, s = sarcomatoid, m = medullary, LN = lymph node, MSKCI/UISS = Memorial Sloan Kettering Cancer Institute/UCLA Integrated Staging System, L = Low, Int = Intermediate
(DOCX)

Table S2 MSigDB Genesets associated with FoxP3, CTLA-4 and TGFß.
(DOCX)

Table S3 Gene Sets enriched in PBL of mRCC patients at an FDR<0.2.
(DOCX)
