# Supplementary figures and images for: Regulatory T-Cells and Associated Pathways in Metastatic Renal Cell Carcinoma (mRCC) Patients Undergoing DC-Vaccination and Cytokine-Therapy
Source: PLoS One. 2012 Oct 31;7(10):e46600. doi: 10.1371/journal.pone.0046600 (PMC3485261; doi:10.1371/journal.pone.0046600)

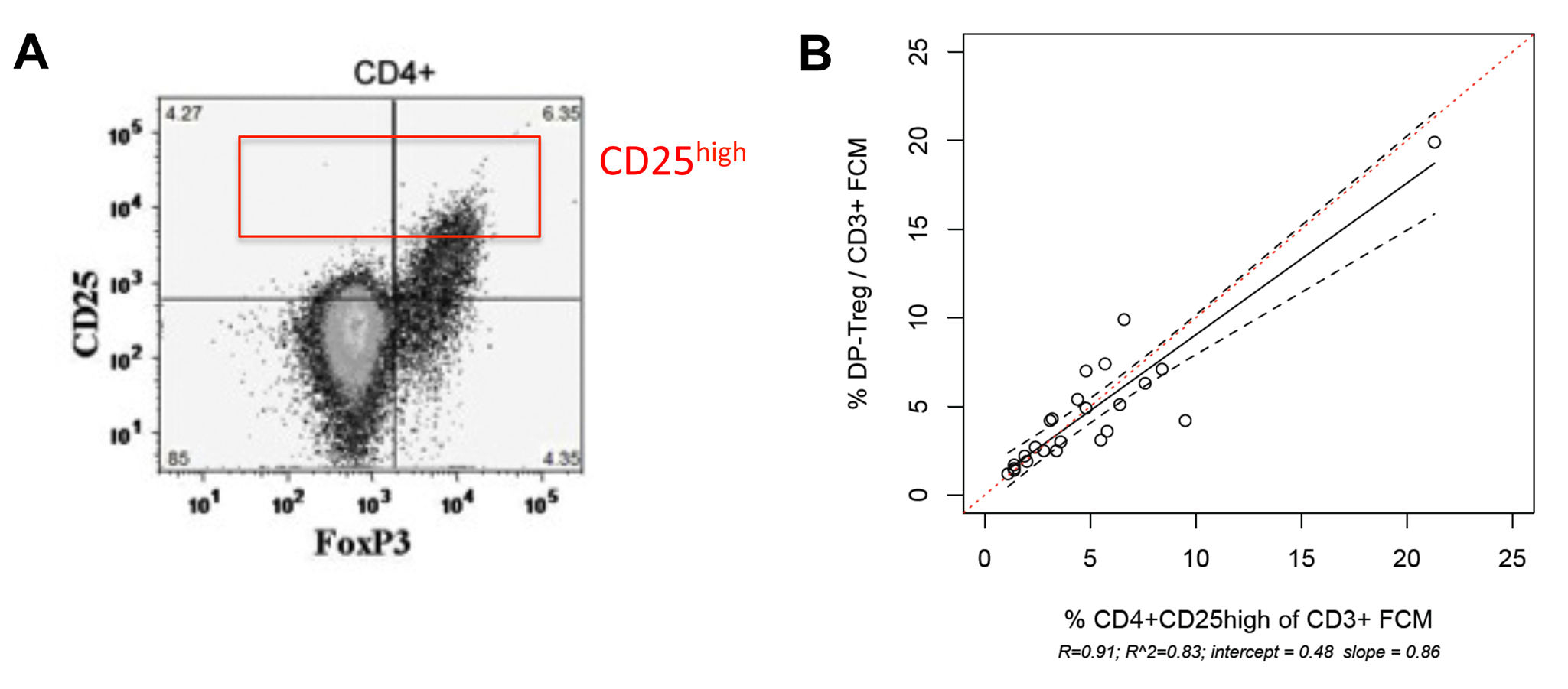

Supplement: Figure S1 — The FCM plot, pre-gated on lymphocytes, shows that within the CD25high population nearly 100% of the cells are also FoxP3+(A), Linear regression between CD4+CD25high T-cells and CD4+CD25+FOXP3+ DP-TREG both measured by Flow Cytometry reveals a correlation of R = 0.91 between the two different populations(B). (TIF) [file pone.0046600.s001.tif]

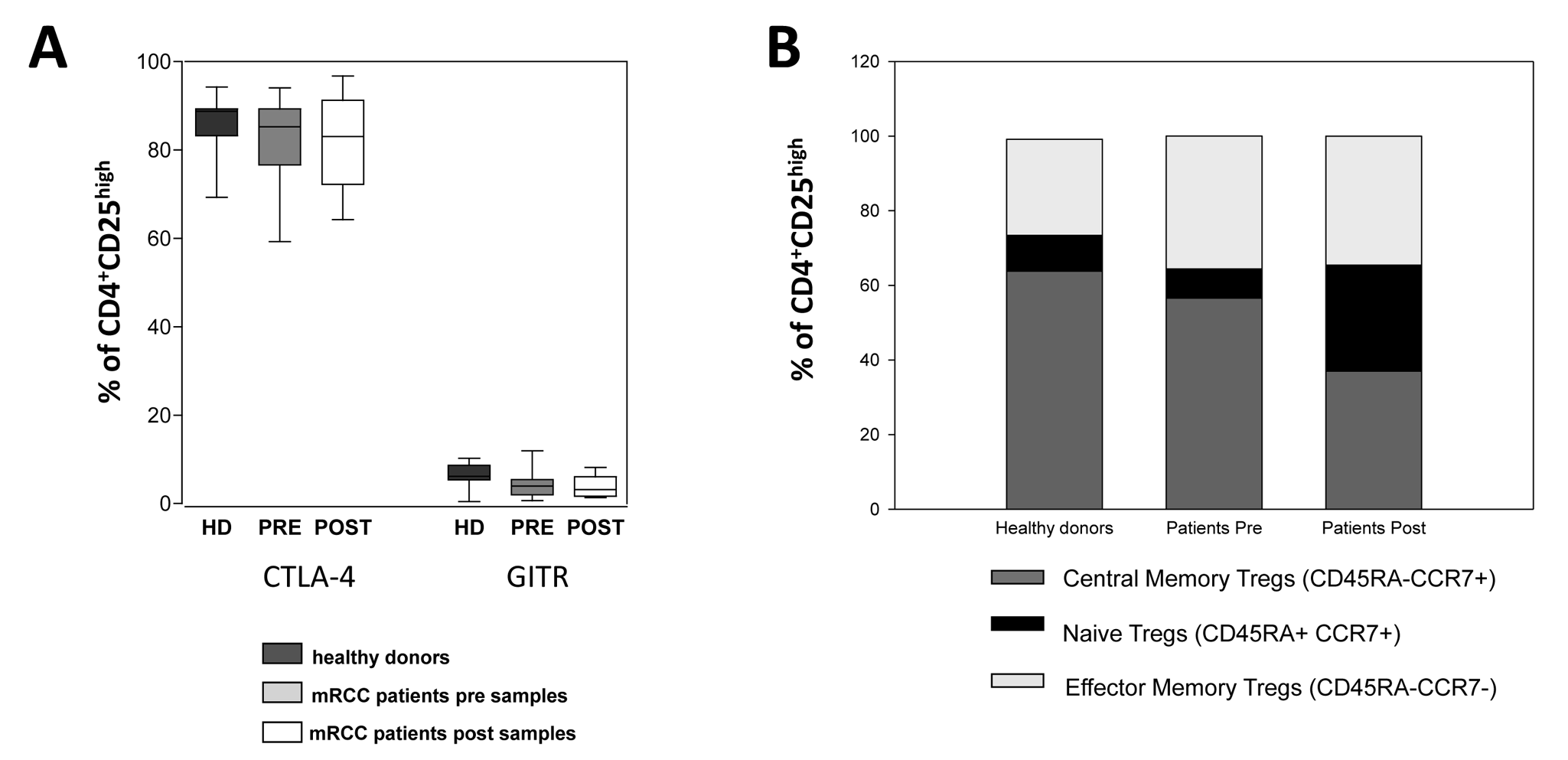

Supplement: Figure S2 — Expression of surface molecules within the CD4+CD25high compartment. (A) Expression of CTLA-4, and GITR did not differ between healthy controls and mRCC patients. (B) Distribution of T-cells belonging to central memory, effector memory or naive phenotype within the CD4+CD25high T-Cell compartment in HD and mRCC patients before and after therapy. (TIF) [file pone.0046600.s002.tif]

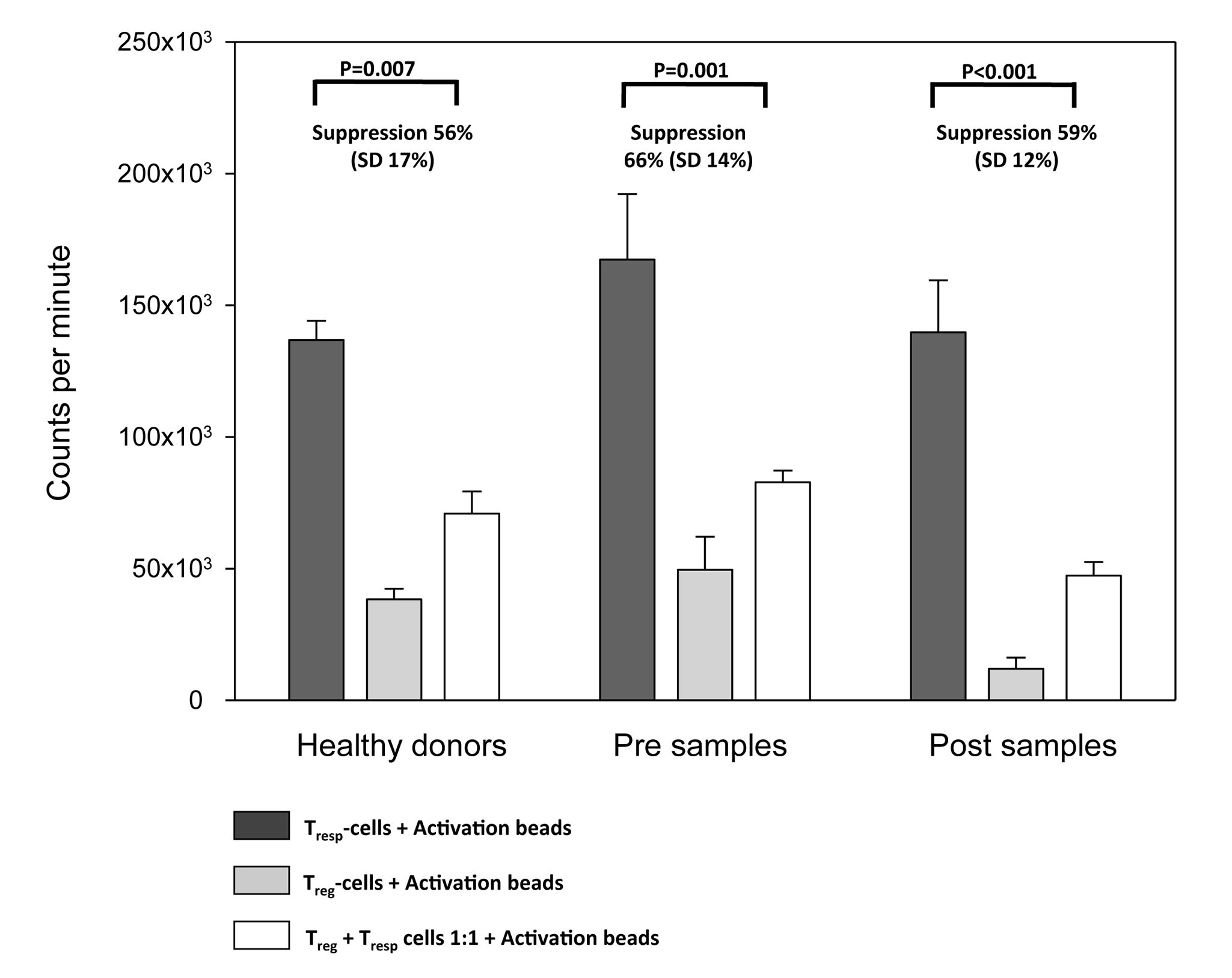

Supplement: Figure S3 — TREG suppression assay. CD4+CD25− T-cells were mixed with CD4+CD25high regulatory T-cells and stimulated with T-cell activation/expansion beads. Proliferation was measured by [3H]-Thymidine incorporation. Patients with pre and post samples available for the assay were: #1,2,5,9,13,16. (TIF) [file pone.0046600.s003.tif]

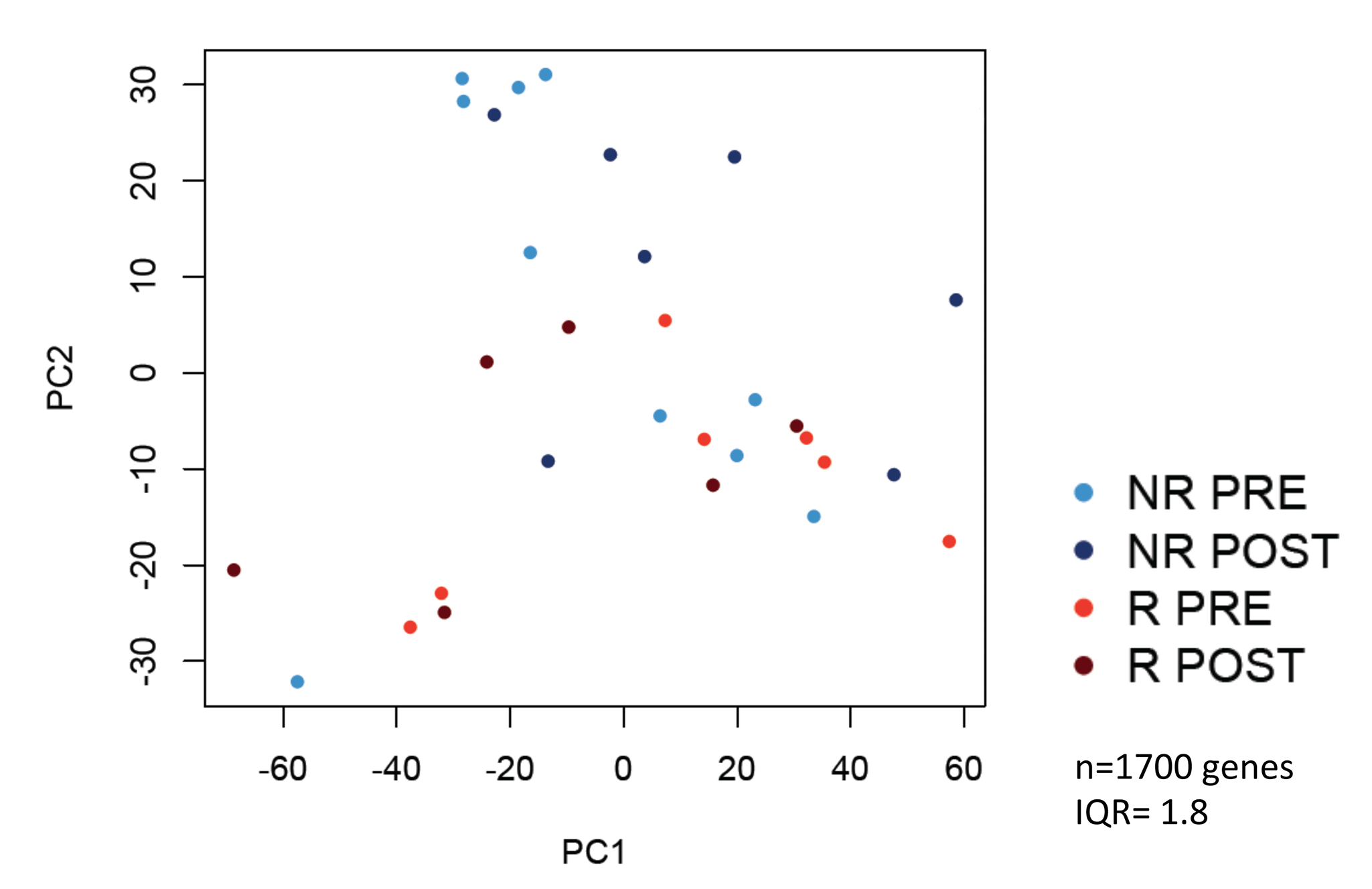

Supplement: Figure S4 — Principle components analysis of the patients' microarray samples with respect to the variables Non-responder, Responder, Pre and Post (TIF) [file pone.0046600.s004.tif]

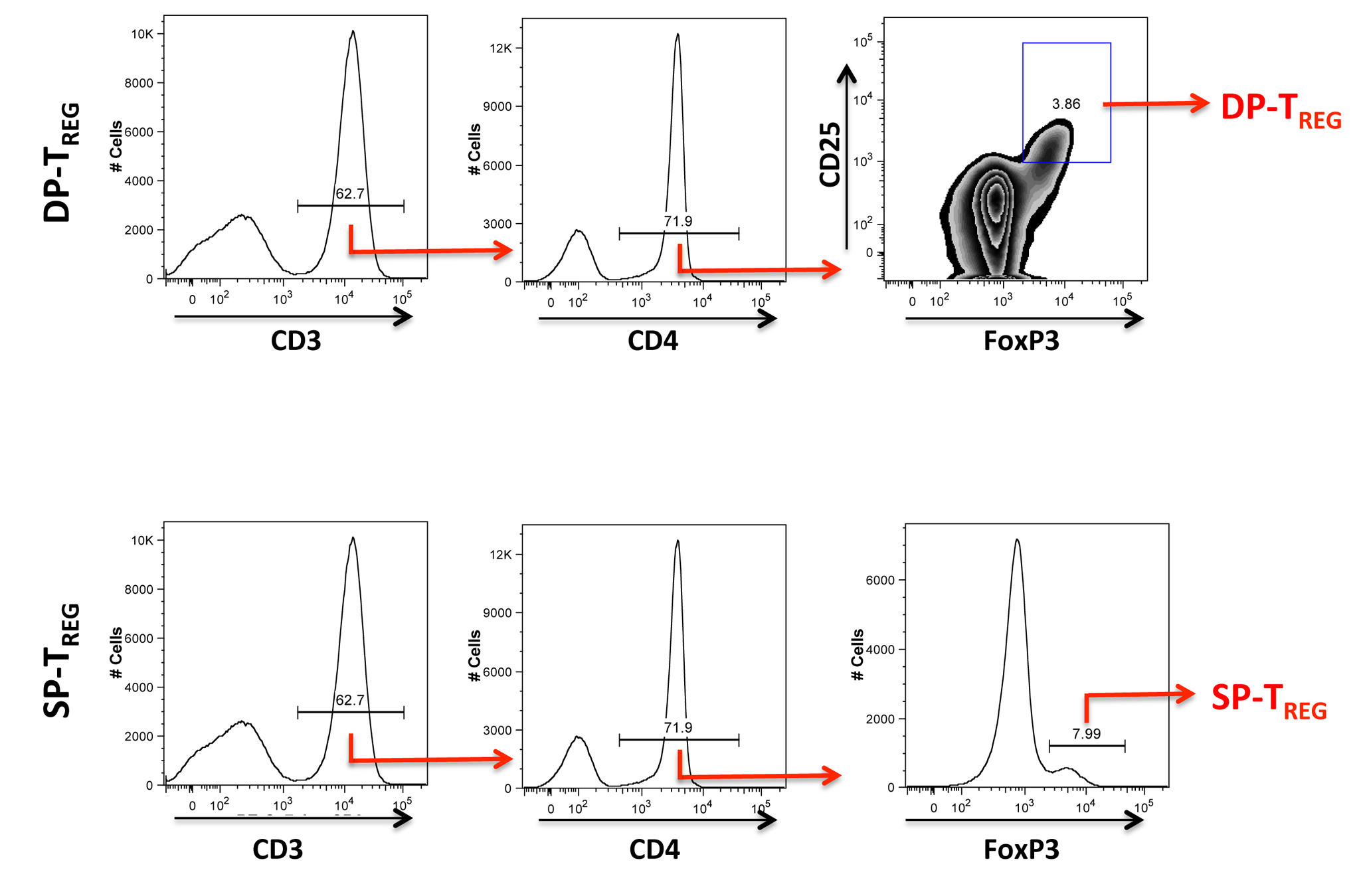

Supplement: Figure S5 — FCM gating strategies for (A) DP- and (B) SP-TREG as described in materials and methods. CD3 plot is pre-gated on lymphocytes by scatter. (TIF) [file pone.0046600.s005.tif]

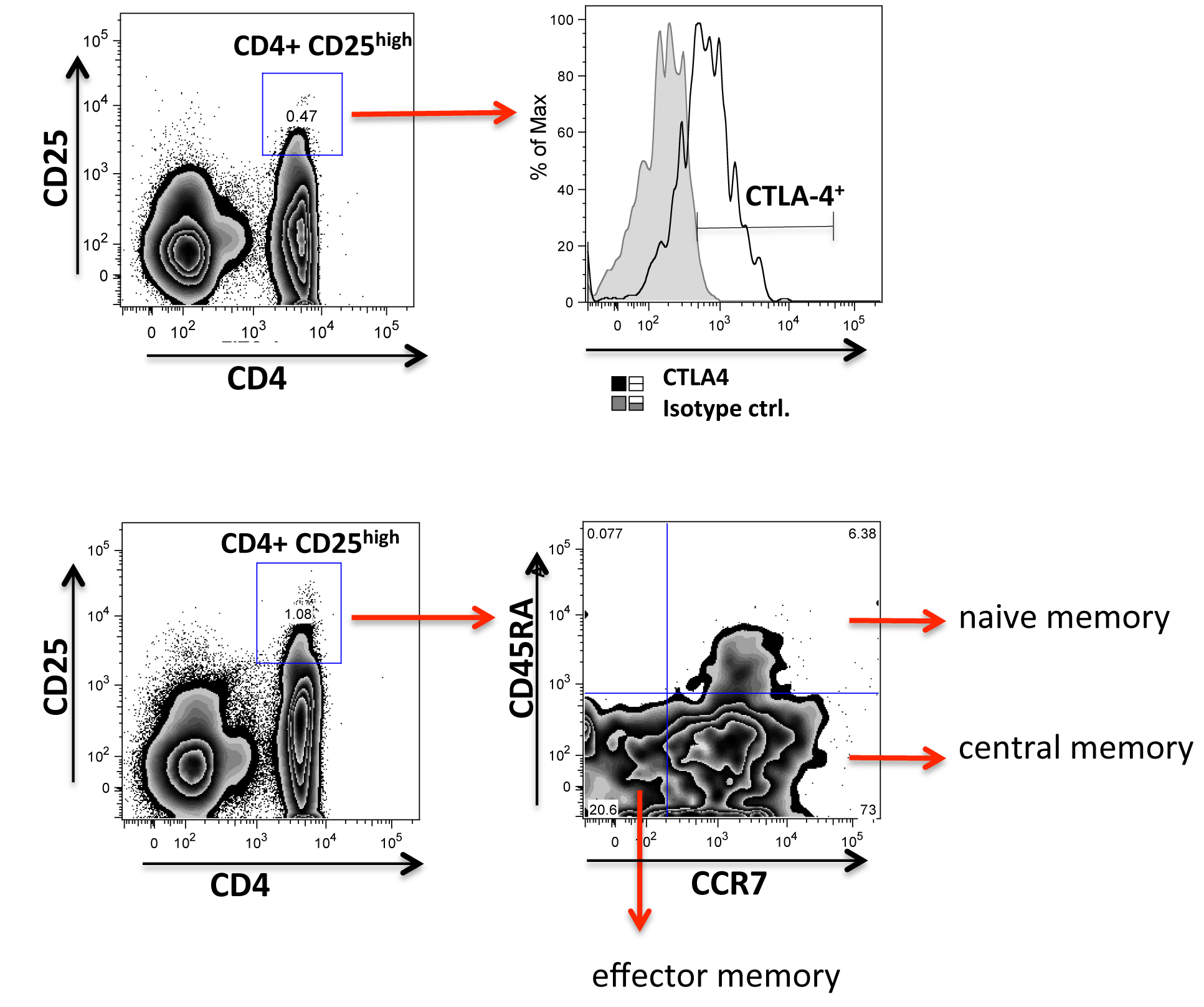

Supplement: Figure S6 — FCM gating strategies for CTLA4: isotype control in gray, CTLA4 Ab solid black line(A), CCR7/CD45RA T memory cell gating strategy(B). CD3 plot is pre-gated on lymphocytes by scatter. (TIF) [file pone.0046600.s006.tif]
