# Supplementary material for: Regulatory T-Cells and Associated Pathways in Metastatic Renal Cell Carcinoma (mRCC) Patients Undergoing DC-Vaccination and Cytokine-Therapy
Source: PLoS One. 2012 Oct 31;7(10):e46600. doi: 10.1371/journal.pone.0046600 (PMC3485261; doi:10.1371/journal.pone.0046600)
Supplement: Table S1 — Clinical data for enrolled patients (n = 18). Abbreviations: M = male, F = female, cc = clear cell, s = sarcomatoid, m = medullary, LN = lymph node, MSKCI/UISS = Memorial Sloan Kettering Cancer Institute/UCLA Integrated Staging System, L = Low, Int = Intermediate (DOCX) [file pone.0046600.s007.docx]

**Table S1:** Clinical data for enrolled patients (n=18)

| **Patient #** | **Gender** | **Age on study** | **Histology** | **Metastatic site** | **MSKCI /UISS** | **Best Overall Response to Treatment** |
| --- | --- | --- | --- | --- | --- | --- |
| 1 | M | 70 | cc | Lung, adrenal | Int/Int | SD: Stable Disease |
| 2 | M | 41 | cc | Lung | Int/Int | PR: Partial Response |
| 3 | M | 76 | cc | LN | L/Int | SD: Stable Disease |
| 4 | M | 64 | cc | Lung, LN | Int/Int | CR: Complete Response |
| 5 | M | 72 | s | Lung | L/Int | PD: Progressive Disease |
| 6 | F | 61 | cc | Bone | L/Int | PD: Progressive Disease |
| 7 | F | 50 | cc | Lung, Adrenal, LN | Int/Int | PR: Partial Response |
| 8 | M | 56 | cc | Lung, bones, LN | Int/Int | CR: Complete Response |
| 9 | M | 69 | cc | Lung, liver, LN | Int/High | PR: Partial Response |
| 10 | M | 64 | cc | Bone, LN | Int/Int | SD: Stable Disease |
| 11 | M | 53 | cc | Lung,LN | L/L | SD: Stable Disease |
| 12 | M | 61 | cc | Lung,LN | L/Int | PR: Partial Response |
| 13 | M | 64 | cc | Lung | L/High | SD: Stable Disease |
| 14 | M | 42 | cc | Lung | Int/Int | SD: Stable Disease |
| 15 | F | 69 | m | Lung,liver | L/Int | SD: Stable Disease |
| 16 | F | 65 | cc | Lung, LN | Int/Int | PR: Partial Response |
| 17 | M | 47 | s | Skin, LN, Lung | Int/Int | CR: Complete Response |
| 18 | F | 64 | cc | Bone, LN, Lung, Adrenal | Int/Int | PD: Progressive Disease |

Abbreviations: M= male, F= female, cc= clear cell, s= sarcomatoid, m= medullary, LN= lymph node, MSKCI/UISS= Memorial Sloan Kettering Cancer Institute/UCLA Integrated Staging System, L= Low, Int= Intermediate
