# Supplementary material for: Regulatory T-Cells and Associated Pathways in Metastatic Renal Cell Carcinoma (mRCC) Patients Undergoing DC-Vaccination and Cytokine-Therapy
Source: PLoS One. 2012 Oct 31;7(10):e46600. doi: 10.1371/journal.pone.0046600 (PMC3485261; doi:10.1371/journal.pone.0046600)
Supplement: Table S2 — MSigDB Genesets associated with FoxP3, CTLA-4 and TGFß. (DOCX) [file pone.0046600.s008.docx]

**Table S2:** MSigDB Genesets associated with FoxP3, CTLA-4 and TGFß

| **MARSON_FOXP3_ TARGETS_ STIMULATED_UP** | **MARSON_FOXP3_ TARGETS_ STIMULATED_DN** | **MARSON_FOXP3_ TARGETS_UP** | **MARSON_FOXP3_ TARGETS_DN** | **BIOCARTA_TCRA_ PATHWAY** | **BIOCARTA_TCR_ PATHWAY** | **BIOCARTA_CTLA4_ PATHWAY** | **BIOCARTA_TGFB_ PATHWAY** | **JAZAG_TGFB1_ SIGNALING_UP** | **JAZAG_TGFB1_ SIGNALING_DN** | **JAZAG_TGFB1_ SIGNALING_VIA_ SMAD4_UP** | **JAZAG_TGFB1_ SIGNALING_VIA_ SMAD4_DN** | **REACTOME_ IMMUNOREGULATORY_INTERACTIONS** | **REACTOME_CTLA4_ INHIBITORY_ SIGNALING** | **V$SMAD4_Q6** | **BIOCARTA_IL2RB_ PATHWAY** |
| --- | --- | --- | --- | --- | --- | --- | --- | --- | --- | --- | --- | --- | --- | --- | --- |
| ITK | LY6A | S100A4 | AA408296 | PTPRC | HRAS | ITK | MAP2K1 | CCDC85B | FGF6 | CGA | CDK17 | IGLV1-40 | PPP2R1B | MEF2C | E2F1 |
| CRTAM | DNTT | S100A6 | ALS2 | CD3G | GRB2 | CD3G | SMAD7 | CRABP2 | CD244 | CDIPT | CDK18 | PVR | PPP2R1A | SLC22A17 | HRAS |
| POU2AF1 | PLIN2 | MALAT1 | CDV3 | CD3D | CD247 | HLA-DRB1 | TGFBR1 | GFER | C5 | GABRB1 | CLDN4 | MICB | LYN | GDF1 | NMI |
| CYTIP | CPEB2 | GM2A | 2410016O06RIK | HLA-DRB1 | ELK1 | CD3D | CREBBP | PTPN22 | RNH1 | OSGIN1 | VAPB | MICA | PPP2R5B | LMO1 | GRB2 |
| ADAM10 | FOXO3 | LRRC8C | BTG1 /// LOC10004735 | CD3E | NFKBIA | CD3E | TGFBR2 | L1CAM | STOML2 | LPAR2 | SPI1 | IGLV1-44 | PPP2R5A | THRA | STAT5A |
| PTGER4 | DNAJB4 | PDLIM2 | PTPN22 | CD247 | NFKB1 | GRB2 | TGFB3 | C14ORF1 | LSR | FGF12 | JAG2 | IGLV5-37 | PPP2R5D | VAPA | STAT5B |
| AI504432 | RAMP1 | HNRPLL | DENND2D | TRA@ | TRB@ | CD247 | SMAD4 | MRPS31 | SUFU | MRPS31 | SYNGR2 | TRB@ | PPP2R5C | LMO4 | FASLG |
| ANXA1 | LTB | PISD /// PISD-PS1 // | ITM2A | TRB@ | FOS | CTLA4 | SMAD3 | IL11 | CCL27 | CRADD | FLT3LG | B2M | CTLA4 | FGF16 | RPS6KB1 |
| TNFSF14 | TEC | SLC2A3 | MBP | FYN | MAP3K1 | TRA@ | SMAD2 | LSM5 | KCNMB1 | CBX5 | CCNE1 | LILRA1 | SRC | FLJ39739 | BCL2L1 |
| PTPN22 |  | TIAM1 | SLFN2 | LCK | SOS1 | TRB@ | CDH1 | VPS4A | GLRX2 | GPR45 | GALR1 | KLRD1 | PTPN11 | RAB1B | AKT1 |
| EEA1 |  | HADH | GCH1 | ZAP70 | RAC1 | PTPN11 | TAB1 | RAPGEF3 | ATP2B2 | GATA4 | PAK3 | CRTAM | AKT1 | CGB7 | FOS |
| MBP |  | RAMP1 | CDT1 | CD4 | PPP3CB | CD86 | TGFB1 | SPON2 | OAZ3 | PGRMC2 | SLC22A6 | CD3G | CD86 | RORA | SOS1 |
| ZFP36L1 |  | ARHGAP9 | SLC29A1 | HLA-DRA | ZAP70 | CD80 | TGFB2 | SLC12A7 | PCGF2 | SRRM1 | NRG1 | CD3D | PDPK1 | IL17RC | BCL2 |
| LPXN |  | NCF4 | BZW1 |  | PIK3CA | ICOS | MAP3K7 | LRRC6 | CSDE1 | EIF1 | TUBA1B | CD3E | CD80 | ZNRF2 | PIK3CA |
| SLC17A6 |  | LOC100045567 /// PNP | ZFP36L2 |  | PPP3CC | LCK | EP300 | PTPRH | PLA1A | CAB39 | LTA | HLA-A | FYN | SFRS6 | IL2RG |
| ZAP70 |  | MYH9 | 2610018G03RIK |  | NFATC4 | PIK3CA | ZFYVE9 | PKIG | TOP2A | SATB1 | S100A1 | IGLV11-55 | PPP2CA | EPC1 | SHC1 |
| TGIF1 |  | SLC9A3R1 | FAM107B |  | SHC1 | PIK3R1 | MAPK3 | HLA-A | EEF1A1 | C5AR1 | AGPAT1 | HLA-C | LCK | WNT1 | FAS |
| TNFRSF19 |  | DDIT4 | IFNG |  | PPP3CA | ICOSLG | SKIL | MED14 | FLRT1 | LBX1 | SCAMP3 | CD40 | PPP2CB | MLL5 | MYC |
| **MARSON_FOXP3_ TARGETS_ STIMULATED_UP** | **MARSON_FOXP3_ TARGETS_ STIMULATED_DN** | **MARSON_FOXP3_ TARGETS_UP** | **MARSON_FOXP3_ TARGETS_DN** | **BIOCARTA_TCRA_ PATHWAY** | **BIOCARTA_TCR_ PATHWAY** | **BIOCARTA_CTLA4_ PATHWAY** | **BIOCARTA_TGFB_ PATHWAY** | **JAZAG_TGFB1_ SIGNALING_UP** | **JAZAG_TGFB1_ SIGNALING_DN** | **JAZAG_TGFB1_ SIGNALING_VIA_ SMAD4_UP** | **JAZAG_TGFB1_ SIGNALING_VIA_ SMAD4_DN** | **REACTOME_ IMMUNOREGULATORY_INTERACTIONS** | **REACTOME_CTLA4_ INHIBITORY_ SIGNALING** | **V$SMAD4_Q6** | **BIOCARTA_IL2RB_ PATHWAY** |
| EVI2B |  | CD81 | ZAP70 |  | NFATC2 | IL2 | APC | LPCAT3 | NAT6 | STK25 | BYSL | HLA-B | YES1 | ERO1LB | PIK3R1 |
| JAK2 |  | MAPRE2 | EMB |  | NFATC3 | HLA-DRA |  | TIMM8B | CWC27 | PKIG | VIL1 | HLA-E | AKT3 | WNT3 | SYK |
| DAP |  | ZFP260 | MYC |  | PIK3R1 | CD28 |  | TIMELESS | MYLPF | UBIAD1 | SF1 | TRA@ | AKT2 | FLI1 | PIK3CG |
| IL2 |  | SNX18 | NFATC1 |  | RASA1 |  |  | CELF2 | ARID3B | C1QB | CYB5A | HLA-G |  | ELOVL5 | CFLAR |
| MLLT3 |  | MCL1 | STX6 |  | NFATC1 |  |  | RNF24 | SAP18 | EP300 | ST6GALNAC2 | HLA-F |  | RALA | PTPN6 |
|  |  | PTPLAD1 | ITK |  | PIK3CG |  |  | PMP22 | SIGMAR1 | CELF2 | TNFRSF10A | HCST |  | PTBP2 | IL2RB |
|  |  | STK10 | POU2AF1 |  | PTPN7 |  |  | CHORDC1 | PCF11 | MRPL48 | DARC | LILRB1 |  | LGI1 | IKZF3 |
|  |  | VIM | POLR1D |  | PRKCA |  |  | NEU3 | P2RX6 | TPPP3 | PKLR | LILRB2 |  | CASKIN2 | IL2RA |
|  |  | D4WSU53E | RNF19A |  | CD3G |  |  | KIF4A | SERPINF2 | CCRN4L | RBMX2 | IGKV1-5 |  | POU2AF1 | SOCS3 |
|  |  | MKNK2 | ENC1 |  | CD3D |  |  | CNIH | PSMC3 | CYP1B1 | PDGFRB | LILRB5 |  | MCRS1 | SOCS1 |
|  |  | PPM1B | GSTT2 |  | MAP2K1 |  |  | STRAP | NAB2 | CNIH | MAPK7 | CD40LG |  | ARID1A | CBL |
|  |  | ITM2C | ETF1 |  | CD3E |  |  | ALDOC | HIST1H2AI | COPZ1 | SNX10 | CD34 |  | IRS1 | RAF1 |
|  |  | TNFRSF4 | M6PR |  | RELA |  |  | FARS2 | GHRL | IGF2BP1 | SDCCAG1 | LILRB3 |  | TMEM25 | BAD |
|  |  | CD24A | FOXP1 |  | MAP2K4 |  |  | PTK7 | USP25 | NPRL2 | LCP2 | LILRB4 |  | DCTN1 | IRS1 |
|  |  | SERINC3 | LOC100046186 /// RAM |  | RAF1 |  |  | DSCR4 | CLCN7 | MYBL2 | PRODH | IGLV7-43 |  | HHEX | MAPK1 |
|  |  | LY6A | RCL1 |  | CYCSP35 |  |  | CCL5 | ABO | IFI35 | CDRT1 | IGLV3-27 |  | COL1A2 | CRKL |
|  |  | RAC2 | TNFSF11 |  | TRA@ |  |  | RASAL2 | ZFP161 | LHX2 | RBP4 | IGKV4-1 |  | PDGFRA | PPIA |
|  |  | PLIN2 | NOLC1 |  | VAV1 |  |  | ITGB7 |  | HCN4 | MFNG | MADCAM1 |  | LAMC2 | MAPK3 |
|  |  | ITGAV | RGS2 |  | PRKCB |  |  | NUP50 |  | TP53I11 | DRD2 | KIR2DL1 |  | FAM38A | JAK1 |
|  |  | CD2 | RNF4 |  | LAT |  |  | SEC23IP |  | RFX4 | ABCD1 | IGLV3-25 |  | AKAP4 | JAK3 |
|  |  | ENO3 | AKR1C18 |  | PLCG1 |  |  | DVL3 |  | CELSR3 | C5 | IGLV3-22 |  | PLA2G2F |  |
|  |  | RCSD1 | DDT |  | FYN |  |  | ABCB8 |  | MYLPF | PTK7 | KIR2DL3 |  | ADAMTS4 |  |
| **MARSON_FOXP3_ TARGETS_ STIMULATED_UP** | **MARSON_FOXP3_ TARGETS_ STIMULATED_DN** | **MARSON_FOXP3_ TARGETS_UP** | **MARSON_FOXP3_ TARGETS_DN** | **BIOCARTA_TCRA_ PATHWAY** | **BIOCARTA_TCR_ PATHWAY** | **BIOCARTA_CTLA4_ PATHWAY** | **BIOCARTA_TGFB_ PATHWAY** | **JAZAG_TGFB1_ SIGNALING_UP** | **JAZAG_TGFB1_ SIGNALING_DN** | **JAZAG_TGFB1_ SIGNALING_VIA_ SMAD4_UP** | **JAZAG_TGFB1_ SIGNALING_VIA_ SMAD4_DN** | **REACTOME_ IMMUNOREGULATORY_INTERACTIONS** | **REACTOME_CTLA4_ INHIBITORY_ SIGNALING** | **V$SMAD4_Q6** | **BIOCARTA_IL2RB_ PATHWAY** |
|  |  | CRIP1 | TGIF1 |  | JUN |  |  | CCPG1 |  | MYO1F | MST1 | CD226 |  | ADAMTSL1 |  |
|  |  | MYO1C | JAK2 |  | LCK |  |  | FSCN2 |  | HGF | KCNMB1 | KIR2DL2 |  | C9ORF27 |  |
|  |  | OSBPL9 | GADD45B |  | MAPK3 |  |  | MAP2K1 |  | FXR2 | CCL27 | KLRC1 |  | ADORA2B |  |
|  |  | TGFBR1 | DUSP6 |  | CALM3 |  |  | SLC6A11 |  | WIPI2 | LIF | KIR3DL1 |  | TNFRSF12A |  |
|  |  | CUGBP2 | IL2 |  | MAPK8 |  |  | EHMT2 |  | MUC6 | ITGB7 | KIR3DL2 |  | SIPA1 |  |
|  |  | EPHX1 |  |  | CALM2 |  |  | SYNGR4 |  | PDZK1IP1 | SHISA5 | KIR2DL4 |  | ABCA1 |  |
|  |  | SAMHD1 |  |  | CALM1 |  |  | EMILIN1 |  | KRT38 | POU2F2 | ITGAL |  | EPHB1 |  |
|  |  | S100A10 |  |  |  |  |  | PDZK1IP1 |  | HDAC4 | RQCD1 | IFITM1 |  | RASAL2 |  |
|  |  | BASP1 /// LOC1000457 |  |  |  |  |  | ATF5 |  | NOSIP | HSPA4 | KLRK1 |  | VDR |  |
|  |  | CYB5 |  |  |  |  |  | GCM2 |  | UBA2 | PHLDA1 | ITGB2 |  | PFN2 |  |
|  |  | RGS16 |  |  |  |  |  | ST8SIA4 |  | HEBP1 | CNKSR1 | ITGB1 |  | HOXA3 |  |
|  |  | ECM1 |  |  |  |  |  | SUPT16H |  | VCX | UNC119 | ITGB7 |  | MEIS2 |  |
|  |  | PSMB8 |  |  |  |  |  | WIF1 |  | GNB2L1 | GUCY2F | IGHV3-23 |  | IL23A |  |
|  |  | PDZK1IP1 |  |  |  |  |  | DAP3 |  | POP7 | BMP2 | PVRL2 |  | FLJ45121 |  |
|  |  | SH3BGRL |  |  |  |  |  | CORIN |  | NAMPT | CD1A | IGLV3-12 |  | HOXA6 |  |
|  |  | CORO1A |  |  |  |  |  | PRDX2 |  | DAXX | NID2 | IGKC |  | HOXA10 |  |
|  |  | CSNK1D |  |  |  |  |  | INSL6 |  | PTMA | CLEC11A | IGLV3-16 |  | LHX4 |  |
|  |  | ETS1 |  |  |  |  |  | WBP4 |  | SPINK5 | AIM2 | TYROBP |  | HOXA9 |  |
|  |  | CAPG |  |  |  |  |  | BUD31 |  | SLC23A1 | ITGA6 | IGLV1-36 |  | DIABLO |  |
|  |  | IRF8 |  |  |  |  |  | N6AMT1 |  | PCGF2 | PNOC | IGLV10-54 |  | TCF4 |  |
|  |  | TNK2 |  |  |  |  |  | BAG5 |  | PAX9 | RPL13A | KIR2DS2 |  | PPP2R2B |  |
|  |  | XCL1 |  |  |  |  |  | CCNE1 |  | KLK11 | RASSF1 | KIR2DS1 |  | TRAF3 |  |
| **MARSON_FOXP3_ TARGETS_ STIMULATED_UP** | **MARSON_FOXP3_ TARGETS_ STIMULATED_DN** | **MARSON_FOXP3_ TARGETS_UP** | **MARSON_FOXP3_ TARGETS_DN** | **BIOCARTA_TCRA_ PATHWAY** | **BIOCARTA_TCR_ PATHWAY** | **BIOCARTA_CTLA4_ PATHWAY** | **BIOCARTA_TGFB_ PATHWAY** | **JAZAG_TGFB1_ SIGNALING_UP** | **JAZAG_TGFB1_ SIGNALING_DN** | **JAZAG_TGFB1_ SIGNALING_VIA_ SMAD4_UP** | **JAZAG_TGFB1_ SIGNALING_VIA_ SMAD4_DN** | **REACTOME_ IMMUNOREGULATORY_INTERACTIONS** | **REACTOME_CTLA4_ INHIBITORY_ SIGNALING** | **V$SMAD4_Q6** | **BIOCARTA_IL2RB_ PATHWAY** |
|  |  | NKG7 |  |  |  |  |  | GSR |  | ITIH3 | PTCH1 | ITGA4 |  | AP2M1 |  |
|  |  |  |  |  |  |  |  | KCNQ4 |  | RAMP1 | SH3BP2 | IGLV2-33 |  | EEF1A1 |  |
|  |  |  |  |  |  |  |  | CASP3 |  | SERTAD1 |  | IGLV5-45 |  | KLF7 |  |
|  |  |  |  |  |  |  |  | WISP3 |  | PILRA |  | CD200 |  | MAFB |  |
|  |  |  |  |  |  |  |  | CSF2RB |  | MRPL4 |  | IGHV7-81 |  | ACACA |  |
|  |  |  |  |  |  |  |  | NRG1 |  | RAB4B |  | CD8A |  | AGER |  |
|  |  |  |  |  |  |  |  | MT3 |  | ARHGEF5 |  | CD8B |  | EPHA2 |  |
|  |  |  |  |  |  |  |  | CCNH |  | CNPY3 |  | IGLV4-60 |  | RPS8 |  |
|  |  |  |  |  |  |  |  | SLC22A7 |  | TP53 |  | CXADR |  | SUV39H2 |  |
|  |  |  |  |  |  |  |  | GMEB1 |  | PADI3 |  | CD96 |  | NOTCH3 |  |
|  |  |  |  |  |  |  |  | CNPY3 |  | HN1 |  | RAET1E |  | NTRK3 |  |
|  |  |  |  |  |  |  |  | ERLIN2 |  | GRHPR |  | IGLV2-23 |  | POLD4 |  |
|  |  |  |  |  |  |  |  | TOPORS |  | MBD1 |  | IGLV8-61 |  | SLC25A14 |  |
|  |  |  |  |  |  |  |  | GTF2H2 |  | HMGA1 |  | FCGR3A |  | HOXB1 |  |
|  |  |  |  |  |  |  |  | PIGN |  | GPR37L1 |  | CD200R1 |  | PSMC5 |  |
|  |  |  |  |  |  |  |  | IL12A |  | TOM1 |  | ICAM1 |  | ATP2A2 |  |
|  |  |  |  |  |  |  |  | KPNA6 |  | SRCAP |  | ICAM4 |  | RGS3 |  |
|  |  |  |  |  |  |  |  | CPSF4 |  | PEG3 |  | ICAM2 |  | TRPS1 |  |
|  |  |  |  |  |  |  |  | SRCAP |  | WDR45 |  | ICAM3 |  | LASS1 |  |
|  |  |  |  |  |  |  |  | CDRT1 |  | FKBP8 |  | CD160 |  | CACNA1G |  |
|  |  |  |  |  |  |  |  | ABCD1 |  | APH1A |  | TRBC1 |  | KCTD15 |  |
|  |  |  |  |  |  |  |  | AASS |  | SHFM1 |  | IGLV2-11 |  | DHX40 |  |
|  |  |  |  |  |  |  |  | ABHD2 |  | CDH6 |  | KLRG1 |  | TREML2 |  |
|  |  |  |  |  |  |  |  | SLC35B1 |  | GLRX2 |  | ULBP3 |  | PHF8 |  |
| **MARSON_FOXP3_ TARGETS_ STIMULATED_UP** | **MARSON_FOXP3_ TARGETS_ STIMULATED_DN** | **MARSON_FOXP3_ TARGETS_UP** | **MARSON_FOXP3_ TARGETS_DN** | **BIOCARTA_TCRA_ PATHWAY** | **BIOCARTA_TCR_ PATHWAY** | **BIOCARTA_CTLA4_ PATHWAY** | **BIOCARTA_TGFB_ PATHWAY** | **JAZAG_TGFB1_ SIGNALING_UP** | **JAZAG_TGFB1_ SIGNALING_DN** | **JAZAG_TGFB1_ SIGNALING_VIA_ SMAD4_UP** | **JAZAG_TGFB1_ SIGNALING_VIA_ SMAD4_DN** | **REACTOME_ IMMUNOREGULATORY_INTERACTIONS** | **REACTOME_CTLA4_ INHIBITORY_ SIGNALING** | **V$SMAD4_Q6** | **BIOCARTA_IL2RB_ PATHWAY** |
|  |  |  |  |  |  |  |  | NUDT21 |  | ERCC5 |  | ULBP1 |  | SST |  |
|  |  |  |  |  |  |  |  | SEC22A |  | MPDU1 |  | CD81 |  | LHB |  |
|  |  |  |  |  |  |  |  | HSF2BP |  | CAMK2B |  | ULBP2 |  | FOXI1 |  |
|  |  |  |  |  |  |  |  | TGM5 |  | CD27 |  | IGLV2-18 |  | MYLK |  |
|  |  |  |  |  |  |  |  | KLRA1 |  | ENO1 |  | IGLV4-69 |  | ZNF410 |  |
|  |  |  |  |  |  |  |  | TINF2 |  | ACTB |  | C3 |  | UBE2E1 |  |
|  |  |  |  |  |  |  |  | GTF3C4 |  | PTPRC |  | CD247 |  | NXPH3 |  |
|  |  |  |  |  |  |  |  | EBP |  | NUP153 |  | CDH1 |  | EVX1 |  |
|  |  |  |  |  |  |  |  | PLP1 |  | HS3ST3A1 |  | VCAM1 |  | PRKAG1 |  |
|  |  |  |  |  |  |  |  | GPR75 |  | BZRAP1 |  | GLYCAM1 |  | FASTK |  |
|  |  |  |  |  |  |  |  | IL9 |  | YWHAE |  | IGLV7-46 |  | WASF2 |  |
|  |  |  |  |  |  |  |  | CAPN9 |  | OR10H2 |  | AMICA1 |  | RHOQ |  |
|  |  |  |  |  |  |  |  | ILF3 |  | LSP1 |  | TRBV12-3 |  | AMMECR1 |  |
|  |  |  |  |  |  |  |  | RGS17 |  | TOM1L1 |  | SELL |  | NFKB2 |  |
|  |  |  |  |  |  |  |  | PWP1 |  | C7ORF16 |  | CD19 |  | CXADR |  |
|  |  |  |  |  |  |  |  | PRELID1 |  | TIAF1 |  | FCGR2B |  | CBFA2T3 |  |
|  |  |  |  |  |  |  |  | YAF2 |  | CDK20 |  | IGLC2 |  | VGF |  |
|  |  |  |  |  |  |  |  | C7ORF16 |  | COG2 |  | IGLC1 |  | PAX1 |  |
|  |  |  |  |  |  |  |  | FEZ2 |  |  |  | IGLC3 |  | TENC1 |  |
|  |  |  |  |  |  |  |  | ATP6V0A2 |  |  |  | IGLV4-3 |  | RTN3 |  |
|  |  |  |  |  |  |  |  |  |  |  |  |  |  | TRIM3 |  |
|  |  |  |  |  |  |  |  |  |  |  |  |  |  | TRIM8 |  |
|  |  |  |  |  |  |  |  |  |  |  |  |  |  | PCBP4 |  |
| **MARSON_FOXP3_ TARGETS_ STIMULATED_UP** | **MARSON_FOXP3_ TARGETS_ STIMULATED_DN** | **MARSON_FOXP3_ TARGETS_UP** | **MARSON_FOXP3_ TARGETS_DN** | **BIOCARTA_TCRA_ PATHWAY** | **BIOCARTA_TCR_ PATHWAY** | **BIOCARTA_CTLA4_ PATHWAY** | **BIOCARTA_TGFB_ PATHWAY** | **JAZAG_TGFB1_ SIGNALING_UP** | **JAZAG_TGFB1_ SIGNALING_DN** | **JAZAG_TGFB1_ SIGNALING_VIA_ SMAD4_UP** | **JAZAG_TGFB1_ SIGNALING_VIA_ SMAD4_DN** | **REACTOME_ IMMUNOREGULATORY_INTERACTIONS** | **REACTOME_CTLA4_ INHIBITORY_ SIGNALING** | **V$SMAD4_Q6** | **BIOCARTA_IL2RB_ PATHWAY** |
|  |  |  |  |  |  |  |  |  |  |  |  |  |  | PAK3 |  |
|  |  |  |  |  |  |  |  |  |  |  |  |  |  | POU4F3 |  |
|  |  |  |  |  |  |  |  |  |  |  |  |  |  | LOXL3 |  |
|  |  |  |  |  |  |  |  |  |  |  |  |  |  | FOXB1 |  |
|  |  |  |  |  |  |  |  |  |  |  |  |  |  | KIRREL3 |  |
|  |  |  |  |  |  |  |  |  |  |  |  |  |  | FGF4 |  |
|  |  |  |  |  |  |  |  |  |  |  |  |  |  | NR2F1 |  |
|  |  |  |  |  |  |  |  |  |  |  |  |  |  | SSBP3 |  |
|  |  |  |  |  |  |  |  |  |  |  |  |  |  | AVP |  |
|  |  |  |  |  |  |  |  |  |  |  |  |  |  | HYAL2 |  |
|  |  |  |  |  |  |  |  |  |  |  |  |  |  | ZNF282 |  |
|  |  |  |  |  |  |  |  |  |  |  |  |  |  | LDB1 |  |
|  |  |  |  |  |  |  |  |  |  |  |  |  |  | OTX2 |  |
|  |  |  |  |  |  |  |  |  |  |  |  |  |  | ARHGEF19 |  |
|  |  |  |  |  |  |  |  |  |  |  |  |  |  | CDK6 |  |
|  |  |  |  |  |  |  |  |  |  |  |  |  |  | COL25A1 |  |
|  |  |  |  |  |  |  |  |  |  |  |  |  |  | CACNG3 |  |
|  |  |  |  |  |  |  |  |  |  |  |  |  |  | CLC |  |
|  |  |  |  |  |  |  |  |  |  |  |  |  |  | PURA |  |
|  |  |  |  |  |  |  |  |  |  |  |  |  |  | MMP11 |  |
|  |  |  |  |  |  |  |  |  |  |  |  |  |  | DOK1 |  |
|  |  |  |  |  |  |  |  |  |  |  |  |  |  | DHRS3 |  |
|  |  |  |  |  |  |  |  |  |  |  |  |  |  | CA9 |  |
|  |  |  |  |  |  |  |  |  |  |  |  |  |  | GNB2 |  |
|  |  |  |  |  |  |  |  |  |  |  |  |  |  | CHRM1 |  |
| **MARSON_FOXP3_ TARGETS_ STIMULATED_UP** | **MARSON_FOXP3_ TARGETS_ STIMULATED_DN** | **MARSON_FOXP3_ TARGETS_UP** | **MARSON_FOXP3_ TARGETS_DN** | **BIOCARTA_TCRA_ PATHWAY** | **BIOCARTA_TCR_ PATHWAY** | **BIOCARTA_CTLA4_ PATHWAY** | **BIOCARTA_TGFB_ PATHWAY** | **JAZAG_TGFB1_ SIGNALING_UP** | **JAZAG_TGFB1_ SIGNALING_DN** | **JAZAG_TGFB1_ SIGNALING_VIA_ SMAD4_UP** | **JAZAG_TGFB1_ SIGNALING_VIA_ SMAD4_DN** | **REACTOME_ IMMUNOREGULATORY_INTERACTIONS** | **REACTOME_CTLA4_ INHIBITORY_ SIGNALING** | **V$SMAD4_Q6** | **BIOCARTA_IL2RB_ PATHWAY** |
|  |  |  |  |  |  |  |  |  |  |  |  |  |  | NAB2 |  |
|  |  |  |  |  |  |  |  |  |  |  |  |  |  | EIF4A1 |  |
|  |  |  |  |  |  |  |  |  |  |  |  |  |  | KCNH6 |  |
|  |  |  |  |  |  |  |  |  |  |  |  |  |  | SNX12 |  |
|  |  |  |  |  |  |  |  |  |  |  |  |  |  | FKBP10 |  |
|  |  |  |  |  |  |  |  |  |  |  |  |  |  | CLOCK |  |
|  |  |  |  |  |  |  |  |  |  |  |  |  |  | PRKCZ |  |
|  |  |  |  |  |  |  |  |  |  |  |  |  |  | YPEL1 |  |
|  |  |  |  |  |  |  |  |  |  |  |  |  |  | USP2 |  |
|  |  |  |  |  |  |  |  |  |  |  |  |  |  | CALD1 |  |
|  |  |  |  |  |  |  |  |  |  |  |  |  |  | POLR2I |  |
|  |  |  |  |  |  |  |  |  |  |  |  |  |  | NR1D1 |  |
|  |  |  |  |  |  |  |  |  |  |  |  |  |  | FMO2 |  |
|  |  |  |  |  |  |  |  |  |  |  |  |  |  | WDR13 |  |
|  |  |  |  |  |  |  |  |  |  |  |  |  |  | PPP3CB |  |
|  |  |  |  |  |  |  |  |  |  |  |  |  |  | NFATC4 |  |
|  |  |  |  |  |  |  |  |  |  |  |  |  |  | PPP3CA |  |
|  |  |  |  |  |  |  |  |  |  |  |  |  |  | ETV4 |  |
|  |  |  |  |  |  |  |  |  |  |  |  |  |  | BAHD1 |  |
|  |  |  |  |  |  |  |  |  |  |  |  |  |  | MAF |  |
|  |  |  |  |  |  |  |  |  |  |  |  |  |  | TBX6 |  |
|  |  |  |  |  |  |  |  |  |  |  |  |  |  | FLRT1 |  |
|  |  |  |  |  |  |  |  |  |  |  |  |  |  | CREB1 |  |
|  |  |  |  |  |  |  |  |  |  |  |  |  |  | TBX5 |  |
|  |  |  |  |  |  |  |  |  |  |  |  |  |  | SLC12A5 |  |
| **MARSON_FOXP3_ TARGETS_ STIMULATED_UP** | **MARSON_FOXP3_ TARGETS_ STIMULATED_DN** | **MARSON_FOXP3_ TARGETS_UP** | **MARSON_FOXP3_ TARGETS_DN** | **BIOCARTA_TCRA_ PATHWAY** | **BIOCARTA_TCR_ PATHWAY** | **BIOCARTA_CTLA4_ PATHWAY** | **BIOCARTA_TGFB_ PATHWAY** | **JAZAG_TGFB1_ SIGNALING_UP** | **JAZAG_TGFB1_ SIGNALING_DN** | **JAZAG_TGFB1_ SIGNALING_VIA_ SMAD4_UP** | **JAZAG_TGFB1_ SIGNALING_VIA_ SMAD4_DN** | **REACTOME_ IMMUNOREGULATORY_INTERACTIONS** | **REACTOME_CTLA4_ INHIBITORY_ SIGNALING** | **V$SMAD4_Q6** | **BIOCARTA_IL2RB_ PATHWAY** |
|  |  |  |  |  |  |  |  |  |  |  |  |  |  | UBE4B |  |
|  |  |  |  |  |  |  |  |  |  |  |  |  |  | DLGAP4 |  |
|  |  |  |  |  |  |  |  |  |  |  |  |  |  | PHF12 |  |
|  |  |  |  |  |  |  |  |  |  |  |  |  |  | SC65 |  |
|  |  |  |  |  |  |  |  |  |  |  |  |  |  | DUSP5 |  |
|  |  |  |  |  |  |  |  |  |  |  |  |  |  | GBA2 |  |
|  |  |  |  |  |  |  |  |  |  |  |  |  |  | CDH13 |  |
|  |  |  |  |  |  |  |  |  |  |  |  |  |  | RPS6KA3 |  |
|  |  |  |  |  |  |  |  |  |  |  |  |  |  | DACH2 |  |
|  |  |  |  |  |  |  |  |  |  |  |  |  |  | BNC2 |  |
|  |  |  |  |  |  |  |  |  |  |  |  |  |  | SULF1 |  |
|  |  |  |  |  |  |  |  |  |  |  |  |  |  | KCNN2 |  |
|  |  |  |  |  |  |  |  |  |  |  |  |  |  | ATP6V1E2 |  |
|  |  |  |  |  |  |  |  |  |  |  |  |  |  | SP8 |  |
|  |  |  |  |  |  |  |  |  |  |  |  |  |  | PBX3 |  |
|  |  |  |  |  |  |  |  |  |  |  |  |  |  | CRK |  |
|  |  |  |  |  |  |  |  |  |  |  |  |  |  | APBB1 |  |
|  |  |  |  |  |  |  |  |  |  |  |  |  |  | CALM1 |  |
