# Supplementary material for: Regulatory T-Cells and Associated Pathways in Metastatic Renal Cell Carcinoma (mRCC) Patients Undergoing DC-Vaccination and Cytokine-Therapy
Source: PLoS One. 2012 Oct 31;7(10):e46600. doi: 10.1371/journal.pone.0046600 (PMC3485261; doi:10.1371/journal.pone.0046600)
Supplement: Table S3 — Gene Sets enriched in PBL of mRCC patients at an FDR<0.2. (DOCX) [file pone.0046600.s009.docx]

**Table S3:** Gene Sets enriched in PBL of mRCC patients at an FDR < 0.2

|  | **NAME** | **SIZE** | **ES** | **NES** | **NOM p-val** | **FDR q-val** | **FWER p-val** | **RANK AT MAX** | **LEADING EDGE** |
| --- | --- | --- | --- | --- | --- | --- | --- | --- | --- |
| **1** | **BIOCARTA _TGFB _PATHWAY** | **19** | **-0.6994** | **-1.4092** | **0.0133** | **0.1735** | **0.0910** | **3935** | **tags=63%, list=20%, signal=79%** |
| **2** | **MARSON_FOXP3 _TARGETS _STIMULATED_UP** | **22** | **-0.7244** | **-1.3705** | **0.0373** | **0.1440** | **0.1355** | **1501** | **tags=55%, list=8%, signal=59%** |
| **3** | **MARSON_FOXP3 _TARGETS _UP** | **54** | **-0.6538** | **-1.3326** | **0.0379** | **0.1725** | **0.2045** | **4010** | **tags=61%, list=20%, signal=76%** |
| **4** | **BIOCARTA _IL2RB _PATHWAY** | **38** | **-0.7233** | **-1.3289** | **0.0344** | **0.1372** | **0.2130** | **3623** | **tags=74%, list=18%, signal=90%** |
| **5** | **BIOCARTA _CTLA4 _PATHWAY** | **18** | **-0.7636** | **-1.3142** | **0.0378** | **0.1340** | **0.2405** | **3217** | **tags=78%, list=16%, signal=93%** |
| **6** | **BIOCARTA_TCR _PATHWAY** | **44** | **-0.7163** | **-1.3008** | **0.0378** | **0.1334** | **0.2655** | **3905** | **tags=75%, list=20%, signal=93%** |
| **7** | **REACTOME _CTLA4 _INHIBITORY _SIGNALING** | **21** | **-0.6727** | **-1.2875** | **0.0444** | **0.1379** | **0.2940** | **4697** | **tags=71%, list=24%, signal=94%** |
